# Total chemical synthesis of pentameric cholera toxin subunit B

**DOI:** 10.1039/d5cc06935a

**Published:** 2026-03-27

**Authors:** Paul Spaltenstein, Samuel R. Scherer, Tyler E. Jones, Riley J. Giesler, Michael S. Kay

**Affiliations:** a Department of Biochemistry, University of Utah 15 North Medical Drive East, Room 4100 Salt Lake City UT 84112 USA kay@biochem.utah.edu

## Abstract

*Vibrio cholerae* annually infects millions worldwide, causing intense and life-threatening diarrheal disease. The pentameric cholera toxin subunit B (CtxB) binds to the ganglioside GM1 on epithelial cells, initiating endocytosis and downstream toxicity. We present the total chemical protein synthesis of l-CtxB and mirror-image d-CtxB using a three-segment N-to-C native chemical ligation approach. Oxidative folding produced the desired 62 kDa pentameric proteins, with l-CtxB capable of binding native GM1 ligand. Synthetic d-CtxB is now ready to serve as a target in mirror-image phage display, representing a significant milestone for the discovery of toxin-neutralizing d-peptides to treat and prevent cholera.

## Abstract 2

*Vibrio cholerae* annually infects millions worldwide, causing intense and life-threatening diarrheal disease.

## Graphical abstract

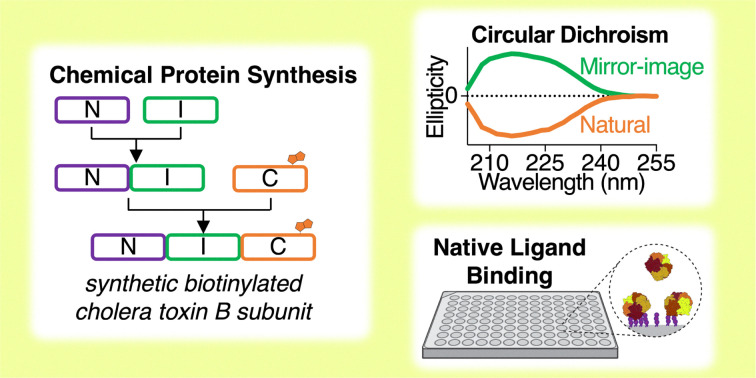

*Vibrio cholerae* annually infects >2.5 million people, mostly in the developing world where sanitation is poor or when access to clean water is limited following disasters.^1^ Toxicity is mediated by cholera toxin (Ctx), comprised of the homopentameric subunit B (CtxB) and the toxic subunit A (CtxA).^2^ CtxB binds monosialoganglioside (GM1) on intestinal epithelial host-cell surfaces, where the toxin is endocytosed and undergoes retrograde trafficking to the ER. CtxA releases, retro-translocates to the cytosol, and activates adenylate cyclase. As a result, cAMP levels rise and hyperactivate chloride (Cl^−^) channels, causing an osmotic shift of water into the gut. The resultant diarrhea is life-threatening without treatment and facilitates the spread of cholera.^3^ The standard of care is carefully measured rehydration therapy, which can be very successful.^4^ Still, an estimated 95 000 deaths are attributed to cholera each year.^1^ An oral cholera vaccine is available, though its effectiveness in children is limited,^4^ and the 10-day immunization period limits its ability to control outbreaks. An inexpensive, stable, and orally available drug that directly neutralizes the toxin would have significant impact on cholera treatment.

Peptides that block the CtxB–GM1 interaction have been investigated,^5^ but natural l-peptides are rapidly degraded, particularly in the gut, significantly limiting their therapeutic potential. In contrast, mirror-image d-peptides are intrinsically protease-resistant^6^ and ideally suited for oral delivery to neutralize the toxin in the gut. d-Peptides can be discovered *via* mirror-image phage display (MIPD), in which phage displaying l-peptide sequences are panned over a synthetically made d-protein target.^7–10^ By law of mirror-image symmetry, l-peptides that bind the synthetic d-target will bind equivalently to the natural l-target when synthesized as d-peptides. FDA-approved d-peptides include difelikefalin (CSL Vifor) and etelcalcetide (Amgen). MIPD-derived d-peptides in the clinic include CPT31^11^ and RD2^12^ (ClinicalTrials.gov IDs: NCT04672083 and NCT03944460). The first and often rate-limiting step in the MIPD workflow is the chemical protein synthesis (CPS) of the target, since d-proteins cannot be expressed recombinantly.

CPS grants total atomic control over the construction of peptides and proteins, making it an indispensable tool for accessing protein samples bearing unique modifications such as post-translational modifications^13–15^ and exploring mirror-image biology.^16–18^ CPS is achieved using solid-phase peptide synthesis (SPPS)^19^ to generate segments of up to ∼50 residues that can be chemically stitched together to create a full-length protein. Native chemical ligation (NCL)^20^ is one such method, which joins a C-terminal thioester and an N-terminal Cys residue (which can later be desulfurized if desired)^21^ to create a traceless peptide bond. More recently, flow chemistries have been applied to generate much longer peptide segments and even whole proteins,^9,22–24^ but remain resource-intensive and less accessible to the field.

Here, we describe the chemical synthesis and pentameric assembly of CtxB. We first synthesized the l-protein using a three-segment approach. Peptide segments were generated by SPPS, which required the incorporation of several pseudoproline (ΨPro) dipeptides to prevent on-resin aggregation.^25^ Then, stepwise NCL yielded the full-length monomeric protein. Pentameric assembly was achieved *via* oxidative folding and dialysis to remove denaturant. Liquid chromatography-mass spectrometry (LC-MS), circular dichroism (CD), analytical size-exclusion chromatography (SEC), and GM1-binding ELISA were used to confirm folded, pentameric protein capable of ligand binding. CtxB was then synthesized in mirror-image using d-amino acids and characterized with CD and SEC to validate its suitability as a target for MIPD.

Automated Ligator (Aligator),^26^ a computational tool to predict optimal ligation strategies for CPS, proposed both two- and three-segment ligation strategies for the synthesis of CtxB. The two-segment approach was quickly abandoned due to failed SPPS attempts to make the C-terminal peptide (A46-N103, Fig. S1). The three-segment retrosynthetic plan (Fig. 1) splits the sequence at M37/A38 and L85/C86, both junctions comprised of suitable thioesters with sufficient kinetics and minimal expected side reactions.^27^ The N-terminal segment of CtxB (1; T1-M37) and the internal segment (2; A38-L85) were prepared as peptide hydrazides (–NHNH_2_) for *in situ* conversion to thioesters.^28^ The C-terminal peptide was synthesized as a carboxylic acid (–OH). To enable NCL at M37/A38, a non-native Cys was introduced at the N-terminus of peptide 2 to be restored to native Ala by radical-mediated desulfurization after the first ligation.^29^ Native C9 of peptide 1 was protected from desulfurization with the acetamidomethyl (Acm) group. Since modifications at the C-terminus of CtxB (distal from the GM1-binding interface) are well-tolerated,^30–32^ a biotin tag was added to the C-terminus of the C-terminal segment (3; C86-A108) for immobilization during MIPD and protein detection during GM1-binding ELISAs. This tag was composed of a Gly-Ser-Gly linker, a pre-biotinylated Lys, and an Ala to minimize bulk around the resin. We envisioned that the full-length monomer assembly would require two RP-HPLC purifications. The first would come after NCL at M37/A38 and one-pot desulfurization of C38 to A38, then the second after the second NCL at L85/C86 and one-pot Acm removal at C9. Notably, our approach only involves two ligation reactions instead of the more laborious four reported by Satoh and coworkers for the chemical synthesis of a monomeric glycosylated CtxB mutant.^33^ Moreover, this work marks the first successful synthesis of pentameric l-CtxB, as well as its d-enantiomer, representing a significant milestone toward the development of d-peptides against cholera.

**Fig. 1:**
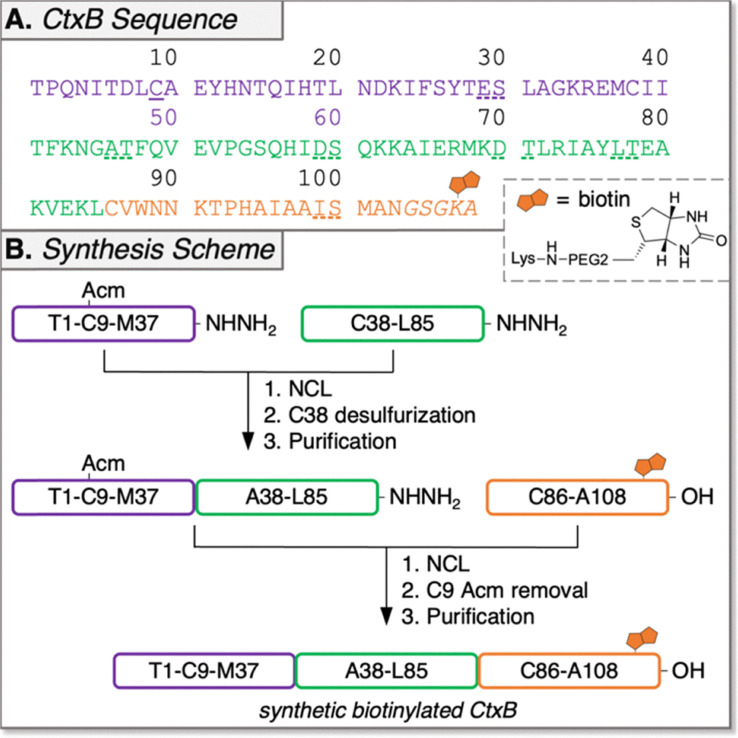
Protein sequence and synthesis scheme for CtxB. (A) CtxB amino acid sequence with peptide segments used for the ligations colored to match section B. Underlined Cys9 residue indicates a native Cys protected with Acm. Bolded position 38 indicates an engineered Cys for NCL that is converted to native Ala *via* desulfurization. Dashed-underlines indicate ΨPro dipeptides. Italicized residues indicate the biotinylated tag. Met residues were substituted with Nle to eliminate oxidation products. (B) Chemical synthesis scheme for biotinylated l- and d-CtxB.

Crude peptide segment quality was greatly improved upon the incorporation of ΨPro dipeptides.^25^ Peptide 1 benefitted from ΨES at position 29–30 and peptide 3 required ΨIS at position 99–100 (Fig. S2). Peptide 2 proved most problematic for achieving appropriate crude peptide quality and required multiple ΨPro (Fig. S3 and S4). Because we planned to repeat the synthesis of CtxB in d-, we aimed to minimize the use of ΨPro to simplify the workflow since d-ΨPro are not commercially available and must be made in-house. We conducted a comprehensive ΨPro screen on peptide 2 and identified that both ΨLT (77–78) and ΨDT (70–71) were required to minimize deletions for the C-terminal half (Q61-L85). We then assessed the need for ΨDS (59–60) and ΨAT (46–47) for the synthesis of the full-length segment (A38-L85) and concluded that both were necessary to achieve desirable crude peptide quality. With optimized SPPS strategies for all three segments, l-peptides were synthesized and purified by RP-HPLC with sufficient purity and yields to proceed with the full-length assembly of l-CtxB (Fig. S5).

The first NCL between l-peptide 1 (1 mM) and l-peptide 2 (1.2 mM) proceeded smoothly over 8 h with minimal thioester hydrolysis and resulted in ligated l-segment 4 (Fig. 2A and Fig. S6). The excess 4-mercaptophenylacetic acid (MPAA), used as the NCL thiol additive, was removed by dialysis to enable one-pot desulfurization since aryl thiols inhibit radical-mediated desulfurization.^34,35^ C38 to A38 desulfurization was achieved in 2 h at 50 °C yielding intermediate l-segment 5, which was purified by RP-HPLC with 34% yield over the one-pot NCL and desulfurization steps (Fig. 2B and Fig. S7). Then, full-length l-CtxB peptide 6 was generated by efficiently ligating l-segment 5 (1.8 mM) with l-peptide 3 (2.7 mM) in 2 h (Fig. 2A and Fig. S8), followed by MPAA removal by dialysis and one-pot Acm removal at C9 with PdCl_2_.^36^ The final l-CtxB product 7 was purified by RP-HPLC with 16% yield over the second NCL and desulfurization step (Fig. 2C and Fig. S9), resulting in a total synthetic yield of 5.4%.

**Fig. 2:**
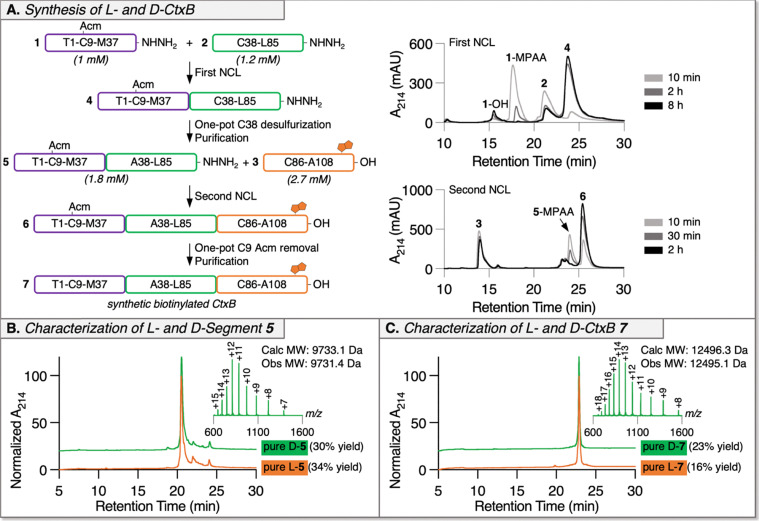
Chemical synthesis of l- and d-CtxB. (A) Analytical RP-HPLC traces of time points for the first ligation between l-peptide 1 and l-peptide 2 and for the second ligation between l-peptide 5 and l-peptide 3. (B) Analytical RP-HPLC (method D) analyses of purified l- and d-intermediate segment 5 along with LC-MS analysis of purified d-segment 5. (C) Analytical RP-HPLC (method D) analyses of purified l- and d-CtxB 7 along with LC-MS analysis of purified d-CtxB 7. Met residues were subsituted with Nle to eliminate oxidation products.

The chemical synthesis of d-CtxB was conducted following the same process as for l-CtxB discussed above. Intermediate d-segment 5 was obtained with 30% yield after the first NCL and one-pot desulfurization. The final d-CtxB product 7 was obtained with 23% yield over the second NCL and desulfurization (Fig. 2 and Fig. S10–S14), leading to a total synthetic yield of 6.9%.

Drawing on our previously reported folding of synthetic Shiga toxin subunit B,^37^ a folding protocol was established using recombinant CtxB. Briefly, recombinant CtxB was denatured in 6 M GnHCl and reduced with 10 mM DTT to mimic the initial state of unfolded and reduced synthetic l- and d-CtxB. Then, refolding was achieved by oxidation with DMSO and stepwise dialysis into PBS. Both LC-MS (to validate disulfide formation) and CD (for secondary structure determination) confirmed that recombinant CtxB properly refolded with a yield of 30% (Fig. S15). This process was repeated with synthetic l- and d-CtxB to access folded protein. Of note, a post-folding purification step by SEC was necessary to remove soluble aggregates, presumably from material that carried synthetic defects preventing proper folding. Purified folded synthetic l- and d-CtxB were analyzed by LC-MS, CD, and SEC. Synthetic l- and d-CtxB have the correct expected molecular weight with successful disulfide formation (Fig. S16). In addition, synthetic l- and d-CtxB have comparable CD spectra to recombinant CtxB with d-CtxB showing the anticipated reciprocal spectrum (Fig. 3A). Analytical SEC analysis revealed comparable elution time and peak shape between l-, d-, and recombinant CtxB (Fig. 3B and Fig. S15). Finally, we validated that synthetic l-CtxB binding to its native receptor GM1 is comparable to recombinant CtxB *via* competitive ELISA. Synthetic l-CtxB (100 nM) was incubated with increasing concentrations of recombinant CtxB (0, 30, 100, 300, and 1000 nM) and added to a microtiter plate coated with GM1. Bound synthetic l-CtxB was detected by probing for biotin using HRP-conjugated streptavidin. The resulting response to increasing concentrations of recombinant CtxB indicates that synthetic l-CtxB is successfully competing for GM1-binding sites with equivalent binding to recombinant CtxB (Fig. 3C). Since mirror-image GM1 is not available, this binding validation could not be performed with d-CtxB, though LC-MS, CD spectroscopy, and SEC analyses confirm comparable folding.

**Fig. 3:**
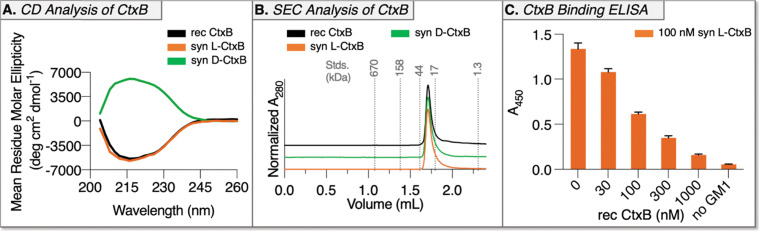
Characterization of folded synthetic l- and d-CtxB. (A) Circular dichroism spectrum of synthetic l-CtxB (orange) matches recombinant CtxB (black) and is reciprocal to the spectrum of synthetic d-CtxB (green). (B) Analytical size-exclusion chromatography traces of synthetic l-, d-, and recombinant CtxB with SEC standards marked. (C) CtxB binding ELISA indicates that synthetic l-CtxB competes for GM1 binding sites with comparable binding to recombinant CtxB. The microtiter plate was coated with 0.0125 ng µL^−1^ GM1. Abbreviations: syn = synthetic; rec = recombinant.

We report the total chemical synthesis of l- and d-CtxB, representing the first major step toward the development of d-peptides to treat and prevent cholera. The synthesis was accomplished using a three-segment N-to-C ligation scheme with a desulfurization step following the first NCL and a final Acm removal to reveal native C9. Importantly, the synthesis was made possible by carefully optimizing the SPPS of the second peptide segment, which required four pseudoproline dipeptides to greatly improve crude quality and access pure material after RP-HPLC purification. Both l- and d-CtxB were folded to the native pentameric state using an oxidative step and stepwise dialysis to remove denaturant. The proteins were characterized by LC-MS, CD spectroscopy, and SEC analyses, revealing successful folding and matched biophysical properties to recombinant CtxB. Furthermore, we demonstrate comparable binding of l-CtxB to recombinant CtxB in a GM1-competition ELISA, ensuring expected function of the synthetic protein.

d-CtxB generated in this study will be subjected to MIPD to identify d-peptides with toxin-neutralizing capabilities. This approach is anticipated to yield a low-cost, easy-to-distribute, and fast-acting orally available peptide therapeutic against cholera.

P. S. and S. R. S. conducted experiments, developed and designed methods, analyzed the data, and wrote the manuscript. T. E. J. and R. J. G. conducted experiments. M. S. K. conceived and supervised the research and acquired funding. All authors contributed to manuscript revisions and editing.

## Conflicts of interest

There are no conflicts to declare.

## Supplementary Material

CC-062-D5CC06935A-s001

## Data Availability

Supporting data can be found in the supplementary information (SI). References for the SI are found in the main text references section.^38,39^ Supplementary information is available. See DOI: https://doi.org/10.1039/d5cc06935a.

## References

[cit1] Deen J., Mengel M. A., Clemens J. D. (2020). Vaccine.

[cit2] Zhang R.-G., Scott D. L., Westbrook M. L., Nance S., Spangler B. D., Shipley G. G., Westbrook E. M. (1995). J. Mol. Biol..

[cit3] Chapman C. M. L., Kapinos A., Rivera-Chávez F. (2023). Infect. Immun..

[cit4] Chowdhury F., Ross A. G., Islam M. T., McMillan N. A. J., Qadri F. (2022). Clin. Microbiol. Rev..

[cit5] Yu R. K., Usuki S., Itokazu Y., Wu H. C. (2016). Glycobiology.

[cit6] Milton R. C. d, Milton S. C. F., Kent S. B. H. (1992). Science.

[cit7] Schumacher T. N., Mayr L. M., Minor Jr D. L., Milhollen M. A., Burgess M. W., Kim P. S. (1996). Science.

[cit8] Qi Y.-K., Zheng J.-S., Liu L. (2024). Chem.

[cit9] Callahan A. J., Gandhesiri S., Travaline T. L., Reja R. M., Lozano Salazar L., Hanna S., Lee Y. C., Li K., Tokareva O. S., Swiecicki J. M., Loas A., Verdine G. L., McGee J. H., Pentelute B. L. (2024). Nat. Commun..

[cit10] Zhou X., Zuo C., Li W., Shi W., Zhou X., Wang H., Chen S., Du J., Chen G., Zhai W., Zhao W., Wu Y., Qi Y., Liu L., Gao Y. (2020). Angew. Chem., Int. Ed..

[cit11] Nishimura Y., Francis J. N., Donau O. K., Jesteadt E., Sadjadpour R., Smith A. R., Seaman M. S., Welch B. D., Martin M. A., Kay M. S. (2020). Proc. Natl. Acad. Sci. U. S. A..

[cit12] van Groen T., Schemmert S., Brener O., Gremer L., Ziehm T., Tusche M., Nagel-Steger L., Kadish I., Schartmann E., Elfgen A., Jürgens D., Willuweit A., Kutzsche J., Willbold D. (2017). Sci. Rep..

[cit13] Lechner C. C., Agashe N. D., Fierz B. (2016). Angew. Chem., Int. Ed..

[cit14] Mali S. M., Singh S. K., Eid E., Brik A. (2017). J. Am. Chem. Soc..

[cit15] Ai H., Chu G. C., Gong Q., Tong Z. B., Deng Z., Liu X., Yang F., Xu Z., Li J. B., Tian C., Liu L. (2022). J. Am. Chem. Soc..

[cit16] Giesler R. J., Woodham P. C. S., Draper S. R. E., Spaltenstein P., Whitby F. G., Hill C. P., Kay M. S. (2025). Chem. Sci..

[cit17] Wang M., Jiang W., Liu X., Wang J., Zhang B., Fan C., Liu L., Pena-Alcantara G., Ling J.-J., Chen J., Zhu T. F. (2019). Chem.

[cit18] Xu Y., Zhu T. F. (2022). Science.

[cit19] Merrifield R. B. (1963). J. Am. Chem. Soc..

[cit20] Dawson P. E., Muir T. W., Clark-Lewis I., Kent S. B. (1994). Science.

[cit21] Yan L. Z., Dawson P. E. (2001). J. Am. Chem. Soc..

[cit22] Hartrampf N., Saebi A., Poskus M., Gates Z. P., Callahan A. J., Cowfer A. E., Hanna S., Antilla S., Schissel C. K., Quartararo A. J., Ye X., Mijalis A. J., Simon M. D., Loas A., Liu S., Jessen C., Nielsen T. E., Pentelute B. L. (2020). Science.

[cit23] Callahan A. J., Rondon A., Reja R. M., Salazar L. L., Gandhesiri S., Rodriguez J., Loas A., Pentelute B. L. (2024). J. Am. Chem. Soc..

[cit24] Yesilcimen A., Gandhesiri S., Travaline T. L., Callahan A. J., Tokareva O. S., Loas A., McGee J. H., Pentelute B. L. (2025). J. Org. Chem..

[cit25] Haack T., Mutter M. (1992). Tetrahedron Lett..

[cit26] Jacobsen M. T., Erickson P. W., Kay M. S. (2017). Bioorg. Med. Chem..

[cit27] Hackeng T. M., Griffin J. H., Dawson P. E. (1999). Proc. Natl. Acad. Sci. U. S. A..

[cit28] Fang G. M., Li Y. M., Shen F., Huang Y. C., Li J. B., Lin Y., Cui H. K., Liu L. (2011). Angew. Chem., Int. Ed..

[cit29] Wan Q., Danishefsky S. J. (2007). Angew. Chem., Int. Ed..

[cit30] Liljeqvist S., Ståhl S., Andréoni C., Binz H., Uhlén M., Murby M. (1997). J. Immunol. Methods.

[cit31] Royal J. M., Oh Y. J., Grey M. J., Lencer W. I., Ronquillo N., Galandiuk S., Matoba N. (2019). FASEB J..

[cit32] Ross J. F., Wildsmith G. C., Johnson M., Hurdiss D. L., Hollingsworth K., Thompson R. F., Mosayebi M., Trinh C. H., Paci E., Pearson A. R., Webb M. E., Turnbull W. B. (2019). J. Am. Chem. Soc..

[cit33] Maki Y., Kawata K., Liu Y., Goo K. Y., Okamoto R., Kajihara Y., Satoh A. (2022). Chem. – Eur. J..

[cit34] Siman P., Blatt O., Moyal T., Danieli T., Lebendiker M., Lashuel H. A., Friedler A., Brik A. (2011). ChemBioChem.

[cit35] Johnson E. C., Kent S. B. (2006). J. Am. Chem. Soc..

[cit36] Maity S. K., Jbara M., Laps S., Brik A. (2016). Angew. Chem., Int. Ed..

[cit37] Fulcher J. M., Petersen M. E., Giesler R. J., Cruz Z. S., Eckert D. M., Francis J. N., Kawamoto E. M., Jacobsen M. T., Kay M. S. (2019). Org. Biomol. Chem..

[cit38] Huang Y. C., Chen C. C., Li S. J., Gao S., Shi J., Li Y. M. (2014). Tetrahedron.

[cit39] Studier F. W. (2005). Protein Expression Purif..

