# Supplementary material for: Total chemical synthesis of pentameric cholera toxin subunit B
Source: Chem Commun (Camb). 2026 Mar 27;62(29):7529–33. doi: 10.1039/d5cc06935a (PMC13023035; doi:10.1039/d5cc06935a)
Supplement: CC-062-D5CC06935A-s001 [file CC-062-D5CC06935A-s001.pdf]

Supplementary Information (SI) for Chemical Communications.
This journal is © The Royal Society of Chemistry 2026

Supporting Information

Total Chemical Synthesis of Pentameric Cholera Toxin Subunit B

Paul Spaltenstein,[†‡] Samuel R. Scherer,[†] Tyler E. Jones, Riley J. Giesler, and Michael S. Kay[*]

Department of Biochemistry, University of Utah, 15 North Medical Drive East, Room 4100, Salt Lake City, UT, USA
* Corresponding Author
† These authors contributed equally.
‡ Current Affiliation: Aliri Bioanalysis, Salt Lake City, UT, USA

SUPPORTING INFORMATION

Sections	Pages
1) Materials	2-3
2) Experimental Procedures	3-13
3) Supplementary Figures	13-25

SECTION 1: Materials

Solid-Phase Peptide Synthesis (SPPS)

Fmoc-L-Ala-OH, Fmoc-L-Cys(Trt)-OH, Fmoc-L-Asp(OtBu)-OH, Fmoc-L-Glu(OtBu)-OH, Fmoc-L-Phe-OH, Fmoc-Gly-OH, Fmoc-L-His(Trt)-OH, Fmoc-L-Ile-OH, Fmoc-L-Lys(Boc)-OH, Fmoc-L-Leu-OH, Fmoc-L-Asn(Trt)-OH, Fmoc-L-Pro-OH, Fmoc-L-Gln(Trt)-OH, Fmoc-L-Arg(Pbf)-OH, Fmoc-L-Ser(tBu)-OH, Fmoc-L-Thr(tBu)-OH, Fmoc-L-Val-OH, Fmoc-L-Trp(Boc)-OH, and Fmoc-L-Tyr(tBu)-OH were purchased from Gyros Protein Technologies and Iris Biotech GmbH. Fmoc-L-Lys(Dde)-OH, Fmoc-D-Lys(Dde)-OH, and Fmoc-L-Norleucine-OH were purchased from AAPPTec. Fmoc-D-Asp(OtBu)-OH, Fmoc-D-Glu(OtBu)-OH, Fmoc-D-His(Trt)-OH, Fmoc-D-Lys(Boc)-OH, Fmoc-D-Asn(Trt)-OH, Fmoc-D-Arg(Pbf)-OH, Fmoc-D-Ser(tBu)-OH, Fmoc-D-Thr(tBu)-OH, Fmoc-D-Val-OH, Fmoc-D-Trp(Boc)-OH, and Fmoc-D-Tyr(tBu)-OH were purchased from GL Biochem. Fmoc-D-Ala-OH, Fmoc-D-Cys(Trt)-OH, Fmoc-D-Ile-OH, and 2-chlorotrityl chloride resin (maximum loading density of 0.77 mmol/g) were purchased from ChemPep. Fmoc-D-Phe-OH and Fmoc-D-Pro-OH were purchased from CBL Patras. Fmoc-D-Leu-OH and Fmoc-D-Gln(Trt)-OH were purchased from AA blocks. Fmoc-D-Norleucine-OH was purchased from Chem-Impex International. Fmoc-Ala-Thr($\Psi^{Me,Me}$pro)-OH, Fmoc-Glu(OtBu)-Ser($\Psi^{Me,Me}$pro)-OH, Fmoc-Asp(OtBu)-Ser($\Psi^{Me,Me}$pro)-OH, Fmoc-Asp(OtBu)-Thr($\Psi^{Me,Me}$pro)-OH, Fmoc-Leu-Thr($\Psi^{Me,Me}$pro)-OH, and Fmoc-Ile-Ser($\Psi^{Me,Me}$pro)-OH were purchased from Iris Biotech GmbH and AAPPTec. Fmoc-D-Ala-D-Thr($\Psi^{Me,Me}$pro)-OH, Fmoc-D-Glu(OtBu)-D-Ser($\Psi^{Me,Me}$pro)-OH, Fmoc-D-Asp(OtBu)-D-Ser($\Psi^{Me,Me}$pro)-OH, Fmoc-D-Asp(OtBu)-D-Thr($\Psi^{Me,Me}$pro)-OH, Fmoc-D-Leu-D-Thr($\Psi^{Me,Me}$pro)-OH, and Fmoc-D-Ile-D-Ser($\Psi^{Me,Me}$pro)-OH were prepared in-house as previously reported.[16] Anhydrous hydrazine (98%), *N,N*-diisopropylethylamine (DIPEA, ReagentPlus grade), piperidine (ReagentPlus grade), *N*-methylmorpholine (NMM, ReagentPlus grade), were purchased from Sigma Aldrich. Fmoc-hydrazine (98%), dimethylformamide (DMF, ACS grade), dichloromethane (DCM, ACS grade), and methanol (MeOH, ACS grade) were purchased from Fisher Scientific. 1-[Bis(dimethylamino)methylene]-1*H*-1,2,3-triazolo[4,5-

b]pyridinium 3-oxid hexafluorophosphate (HATU, 99%) was purchased from Oakwood Chemical. N-Biotinyl-NH-(PEG)$_2$-COOH (biotin-PEG$_2$) was purchased from Novabiochem.

Peptide Cleavage and Precipitation

Trifluoroacetic acid (TFA, peptide synthesis grade) and anhydrous ethyl ether (ACS grade) were purchased from Fisher Scientific. Triisopropylsilane (TIS, 98%) were purchased from Sigma Aldrich. 1,2-ethanedithiol (EDT, 95%) was purchased from Acros Organics.

RP-HPLC and LC-MS

Trifluoroacetic acid (TFA, HPLC grade) was purchased from Alfa Aesar. Acetonitrile (MeCN, HPLC grade), 0.1% formic acid in water (Optima LC-MS grade), and 0.1% formic acid in acetonitrile (Optima LC-MS grade) were purchased from Fisher Scientific.

Ligation, Desulfurization, and Acm Removal

Guanidine hydrochloride (GnHCl, ≥99.5%) was purchased from Thermo Scientific. Sodium phosphate dibasic heptahydrate (ACS grade) was purchased from AMRESCO. Sodium phosphate monobasic (ACS grade), hydrochloric acid (HCl, ACS plus grade), 4-mercaptophenylacetic acid (MPAA, 97%), and sodium nitrite ($NaNO_2$, ACS grade) were purchased from Fisher Scientific. Sodium hydroxide (NaOH, ACS grade), hydrochloric acid (HCl, ACS grade), and acetic acid (>99.8%) were purchased from Acros Organics. Tris (2-carboxyethyl) phosphine hydrochloride (TCEP-HCl, ≥99%) was purchased from Hampton Research. VA-044 was purchased from Wako. L-glutathione reduced (GSH, ≥98%), palladium(II) chloride ($PdCl_2$, ≥99.9%) was purchased from Sigma Aldrich. Dithiothreitol (DTT, ≥99%) was purchased from Gold Biotechnology.

SECTION 2: Experimental Procedures

Automated Peptide Synthesis

All peptides were synthesized on a PurePep Chorus instrument (Gyros Protein Technologies) using Fmoc SPPS. All deprotection and coupling cycles were performed with nitrogen bubbling, and gentle shaking was also used for coupling steps. All cycles were performed at room temperature (r.t.).

Syntheses were conducted at a 25 µmol scale with deprotection cycles consisted of two consecutive 3 min treatments of 2 mL 20% PIP in DMF, followed by three 30 s washes of 2 mL DMF. Coupling cycles consisted of 25

min treatment with a solution of 0.75 mL 200 mM amino acid in DMF, 0.75 mL 195 mM HATU in DMF, and 0.5 mL 600 mM NMM in DMF, followed by three 30 s washes of 2 mL DMF.

Washing, Swelling, and Mixing of Resins

Resins were washed and swelled with approximately 3 mL of specified solvent. The wash volumes for all other synthesis scales are indicated in the relevant sections below. All swelling and manual coupling/deprotection steps were mixed on a rotisserie at r.t.

Preparation of Peptide Resins

To prepare **C-terminal acid** peptides at 25 µmol scale, 150 mg 2-chlorotrityl chloride resin (0.77 mmol/g) was weighed into a 6 mL SPPS tube. The resin was washed three times with DMF, followed by three DCM washes. In order to load the first amino acid onto the resin, 0.03 mmol of the Fmoc-protected amino acid was dissolved in 1 mL of a 1:1 DMF/DCM mixture. 0.15 mmol DIPEA (26 µL) was then mixed into the amino acid solution. The entire amino acid and DIPEA solution was added to the 2-chlorotrityl chloride resin, and the coupling reaction was rotated for 1 h. The resin was then washed three times with DCM. Unreacted 2-chlorotrityl chloride was capped by repeatedly washing the resin with a 17:2:1 DCM:MeOH:DIPEA mixture (~20 mL total used). Once the capping reaction was complete, the resin was washed three times with DCM, followed by three DMF washes. Finally, the resin was transferred to the instrument for automated SPPS. This procedure generates resin with a loading density of approximately 0.2 mmol/g. See "*Determining Resin Loading Density using Fmoc Absorbance"* for more on loading density.

To synthesize **C-terminal peptide-NHNH$_2$** at 25 µmol scale, Fmoc-hydrazine on 2-chlorotrityl chloride resin was prepared based on an established protocol,[38] with several modifications. 150 mg of 2-chlorotrityl chloride resin (0.77 mmol/g) was weighed into a 6 mL SPPS tube. The resin was washed three times with DCM. 1.2 mL DCM was then added to the resin, and the resin was allowed to swell for 10 min at 4 °C. 30.6 µmol Fmoc-hydrazine (7.8 mg) was dissolved in a mixture of 1.5 mL DMF and 0.3 mL DCM, followed by addition of 266 µL DIPEA. Once the 2-chlorotrityl chloride resin was finished swelling, the Fmoc-hydrazine and DIPEA solution was added to the resin slurry at 4 °C. The resin slurry was then placed on a rotisserie at r.t., and the coupling reaction was rotated for 1 h. Once the coupling reaction was finished, 30 µL MeOH was added to the resin (without draining the SPPS tube) in order to cap unreacted 2-chlorotrityl chloride (rotated for 10 min). The resin was then washed three

times with DMF, followed by three DCM washes. The resin was then swelled in a 1:1 DMF/DCM mixture for ≥ 10 min and transferred to the instrument for automated SPPS. This procedure generates resin with a loading density of approximately 0.2 mmol/g. See "*Determining Resin Loading Density using Fmoc Absorbance"* for more on loading density.

Determining Resin Loading Density using Fmoc Absorbance

The resin loading density was determined by Fmoc removal and Fmoc absorbance. To do so, the resin (25 µmol scale) was treated with 4 mL of 20% PIP in DMF with mixing for 20 min. The PIP-Fmoc solution was collected and diluted 100x with 20% PIP in DMF. Triplicate Fmoc absorbance measurements were taken at 280 nm using a NanoDrop One^C instrument (Thermo Scientific) blanked with 20% PIP in DMF. The average A_{280} was used to calculate the Fmoc concentration using an A_{280} extinction coefficient of 5680 $M^{-1}cm^{-1}$. From there, the loading density can be calculated as mmol/g.

Manual Lys(Dde) Deprotection for Biotin-PEG$_2$ Incorporation

For 25 µmol scale Lys(Dde) deprotection, peptide resin was treated with 2 mL 5% hydrazine in DMF for three consecutive 5 min treatments, with mixing. Following the hydrazine treatment, the resin was washed six times with DMF.

Manual Biotin-PEG$_2$ Coupling

Coupling of biotin-PEG$_2$ was completed at 25 µmol scale in 1 h on a rotisserie at r.t. with a solution of 1.5 mL 41 mM biotin-PEG$_2$ in DMF, 0.3 mL 198 mM HATU in DMF, and 0.3 mL 600 mM NMM in DMF, followed by three 30 s washes of 2 mL DMF.

Cleavage and Peptide Precipitation

25 µmol scale peptide cleavage was typically accomplished with a 3 h treatment of 3 mL TFA containing 2.5% water and 2.5% TIS, with mixing on a rotisserie at r.t. For peptides containing Cys(Trt) 2.5% EDT was added to the TFA cocktail. After cleavage, the TFA solution was added to ~30 mL ice-cold ethyl ether, shaken thoroughly, and placed at -20 °C for ≥ 30 min, in order to precipitate the crude peptide. The solution was then centrifuged at 4,700 *g*, 4 °C for ≥ 10 min. The resulting supernatant was decanted, and crude peptide pellets were washed twice with ~20 mL ice-cold ethyl ether. The crude peptide pellets were dried in a vacuum desiccator overnight.

Preparation of Crude Peptides for Analytical RP-HPLC, LC-MS, and Preparative RP-HPLC

25 µmol scale crude peptides were dissolved in 10-20% MeCN 0.1% TFA (~20-40 mL total volume) and were vortexed and sonicated to dissolve as much material as possible, followed by centrifugation at 4,700 *g*, 4 °C for 10 min prior to analytical RP-HPLC, preparative RP-HPLC, and/or LC-MS. Smaller and larger scale sample preparations were conducted by adjusting the amount of crude peptide and solvent adequately.

Analytical LC-MS Methods

0.1% formic acid in water (buffer A) and 0.1% formic acid in MeCN (buffer B) were used as mobile phases for LC-MS analyses. Mass spectra were obtained on an Agilent 6120 single-quadrupole mass spectrometer in fast scan/positive ion mode with an Agilent 1260 Infinity II front-end. UV data were collected using the Agilent 1260 Infinity II diode array detector (200-600 nm). Unless otherwise noted, observed masses were calculated using the charge states from averaged scans across the major ion signal and corresponding UV peak. Calculated and observed masses are presented as average mass. LC-MS methods are described below:

- **LC-MS Method A:** Phenomenex Aeris Widepore 3.6 µm C4 (200 Å, 2.1 x 50 mm) 50 °C; gradient: 0-1 min 5% B, 1-8 min 5-90% B, 8-8.1 min 90-5% B, 8.1-10 min 5% B; flow rate: 0-10 min 0.5 mL/min; scan range: 200-2,000 *m/z*; voltage: 50 V
- **LC-MS Method B:** Phenomenex Aeris Widepore 3.6 µm C4 (200 Å, 2.1 x 50 mm) 50 °C; gradient: 0-1 min 5% B, 1-16 min 5-90% B, 16-16.1 min 90-5% B, 16.1-18 min 5% B; flow rate: 0-18 min 0.5 mL/min; scan range: 400-2,000 *m/z*; voltage: 50 V

Analytical RP-HPLC Methods

0.1% TFA in water (buffer A) and 0.1% TFA in 90% MeCN (buffer B) were used as mobile phases for analytical RP-HPLC analyses. Traces were collected on an Agilent 1260 Infinity II instrument at A_{214}. Analytical RP-HPLC methods are described below:

- **Analytical Method A:** Phenomenex Jupiter 4 µm C12 (90 Å, 4.6 x 150 mm); 40 °C; gradient: 0-2 min 10% B, 2-27 min 10-90% B, 27-30 min 90% B, 30-30.1 min 90-10% B, 30.1-34 min 10% B; flow rate: 1.0 mL/min
- **Analytical Method B:** Phenomenex Jupiter 5 µm C4 (300 Å, 4.6 x 150 mm); 40 °C; gradient: 0-2 min 10% B, 2-5 min 10-20% B, 5-30 min 20-40% B, 30-30.1 min 40-90% B, 30.1-33 min 90% B, 33-33.1 min 90-10% B, 33.1-37 min 10% B; flow rate: 1.0 mL/min

- **Analytical Method C:** Phenomenex Jupiter 5 µm C4 (300 Å, 4.6 x 150 mm); 40 °C; gradient: 0-2 min 10% B, 2-5 min 10-15% B, 5-30 min 15-45% B, 30-30.1 min 45-90% B, 30.1-33 min 90% B, 33-33.1 min 90-10% B, 33.1-37 min 10% B; flow rate: 1.0 mL/min
- **Analytical Method D:** Phenomenex Jupiter 5 µm C4 (300 Å, 4.6 x 150 mm); 40 °C; gradient: 0-2 min 10% B, 2-5 min 10-25% B, 5-30 min 25-55% B, 30-30.1 min 55-90% B, 30.1-33 min 90% B, 33-33.1 min 90-10% B, 33.1-37 min 10% B; flow rate: 1.0 mL/min

Preparative RP-HPLC Methods

0.1% TFA in water (buffer A) and 0.1% TFA in 90% MeCN (buffer B) were used as mobile phases for preparative RP-HPLC purifications. Peptide purifications were performed on an Agilent 1260 Infinity LC system semi-preparative or Agilent 1260 Infinity II preparative system. RP-HPLC purification methods are described below:

- **Purification Method A:** Phenomenex Jupiter 4 µm C12 (90 Å, 21.20 x 250 mm); gradient: 0-4 min 10% B, 4-5 min 10-28% B, 5-30 min 28-34% B, 30-30.1 min 34-90% B, 30.1-34 min 90% B, 34-34.1 min 90-10% B, 34.1-38.5 min 10% B; flow rate: 15.0 mL/min
- **Purification Method B:** Phenomenex Jupiter 4 µm C12 (90 Å, 21.20 x 250 mm); gradient: 0-4 min 20% B, 4-5 min 20-33% B, 5-30 min 33-39% B, 30-30.1 min 39-90% B, 30.1-34 min 90% B, 34-34.1 min 90-20% B, 34.1-38.5 min 20% B; flow rate: 15.0 mL/min
- **Purification Method C:** Phenomenex Jupiter 4 µm C12 (90 Å, 21.20 x 250 mm); gradient: 0-4 min 10% B, 4-5 min 10-25% B, 5-30 min 25-31% B, 30-30.1 min 31-90% B, 30.1-34 min 90% B, 34-34.1 min 90-10% B, 34.1-38.5 min 10% B; flow rate: 15.0 mL/min
- **Purification Method D:** Phenomenex Jupiter 5 µm C4 (300 Å, 10 x 250 mm); 50 °C; gradient: 0-10 min 20% B, 10-10.5 min 20-30% B, 10.5-35.5 min 30-38% B, 35.5-36 min 39-90% B, 36-40 min 90% B, 40-40.1 min 90-20% B, 40.1-45 min 20% B; flow rate: 4.0 mL/min
- **Purification Method E:** Phenomenex Jupiter 5 µm C4 (300 Å, 10 x 250 mm); 50 °C; gradient: 0-10 min 20% B, 10-10.5 min 20-31% B, 10.5-40.5 min 31-40% B, 40.5-41 min 40-90% B, 41-45 min 90% B, 45-45.1 min 90-20% B, 45.1-50 min 20% B; flow rate: 4.0 mL/min

SUPPORTING INFORMATION

Fractions collected during preparative RP-HPLC purifications were analyzed by LC-MS to assess purity. All pure fractions were pooled and lyophilized to obtain dry, pure peptide which was then analyzed by analytical RP-HPLC and LC-MS.

Preparation of Analytical RP-HPLC and LC-MS Traces

Analytical RP-HPLC and LC-MS traces were prepared for publication using our in-house Automated Trace Maker (ATM) programs. Using .CSV files of analytical RP-HPLC or LC-MS data, these Python scripts generate the desired chromatograms that can be viewed in Microsoft Excel. All m/z plots were generated from the entire chromatogram of each run. The ATM programs are available for free use on the Kay Lab Github website: https://github.com/kay-lab.

Calculation of Peptide Concentrations

Peptide concentrations were determined using A_{280} measurements collected on a NanoDrop One^{C} instrument. The following extinction coefficients were used to calculate concentrations via Beer's Law:

- Trp – 5,500 $M^{-1}cm^{-1}$
- Tyr – 1,490 $M^{-1}cm^{-1}$
- Disulfide – 125 $M^{-1}cm^{-1}$

Native Chemical Ligation (NCL) with Peptide 1 and Peptide 2

Peptide 1 was dissolved to 2.5 mM in activation buffer (6 M GnHCl, 100 mM phosphate, pH 3.0) and activated by sodium nitrite treatment (freshly prepared 15 eq $NaNO_2$) for 20 min at -20 °C. Following activation, a solution containing freshly prepared MPAA pH 7.0 in ligation buffer (6 M GnHCl, 100 mM phosphate, pH 7.0) was added to a final concentration of 100 mM, and the pH was adjusted to 6.8 to initiate thiolysis. Peptide 2 was dissolved in ligation buffer to 4 mM and added to the ligation reaction (final concentration of 1.2 mM; 1.2 eq to peptide thioester). After 10 min, TCEP in ligation buffer was added to the reaction to a final concentration of 15 mM and the reaction was left to rotate on a rotisserie at r.t until complete. Time points for the intermolecular NCL reactions were taken by diluting an aliquot of reaction 1:10 with 200 mM TCEP in LC-MS-grade water (pH adjusted to 7.0 prior to addition). Acetic acid was added to a final concentration of ~5% and the dilution was centrifuged at 18,000 *g*, r.t for 10 min prior to analytical RP-HPLC and/or LC-MS analyses. Upon completion (based on analytical RP-HPLC and LC-MS), MPAA was removed by dialyzing against ligation buffer using a 3.5 kDa MWCO

cassette to enable one-pot desulfurization. For the synthesis of D-CtxB, this reaction was performed as described, but with the corresponding D-peptides.

Desulfurization of Peptide 4

"Desulfurization buffer" (6 M GnHCl, 100 mM $NaPO_4$, pH 6.5) was first sparged with argon gas for ≥ 20 min. 400 mM reduced glutathione, 200 mM VA-044 in desulfurization buffer was prepared, along with a solution of 600 mM TCEP in desulfurization buffer. The VA-044/GSH solution was added to a final concentration of 60 mM VA-044 and 120 mM GSH, followed by TCEP to a final concentration of 300 mM. The resulting desulfurization reaction was briefly vortexed and carefully pH adjusted to 6.5 with NaOH. The desulfurization reaction was then covered with argon gas and placed at 50 °C until completion. Time points were taken by first diluting 20 µL desulfurization reaction in 100 µL 20% RP-HPLC buffer B. After vortexing, the dilution was centrifuged at 18,000 *g*, r.t. for 10 min prior to analytical RP-HPLC and LC-MS analyses. The desulfurized product peptide 5 was purified by semi-preparative RP-HPLC. For the synthesis of D-CtxB, this reaction was performed as described, but with the corresponding D-peptide.

Native Chemical Ligation (NCL) with Peptide 5 and Peptide 3

Peptide 5 was dissolved to 4 mM in activation buffer (6 M GnHCl, 100 mM phosphate, pH 3.0) and activated by sodium nitrite treatment (freshly prepared 15 eq $NaNO_2$) for 20 min at -20 °C. Following activation, a solution containing freshly prepared MPAA pH 7.0 in ligation buffer (6 M GnHCl, 100 mM phosphate, pH 7.0) was added to a final concentration of 100 mM, and the pH was adjusted to 6.8 to initiate thiolysis. Peptide 3 was dissolved in ligation buffer to 6 mM and added to the ligation reaction (final concentration of 2.7 mM; 1.5 eq to peptide thioester). After 10 min, TCEP in ligation buffer was added to the reaction to a final concentration of 15 mM and the reaction was left to rotate on a rotisserie at r.t until complete. Time points for the intermolecular NCL reactions were taken by diluting an aliquot of reaction 1:10 with 200 mM TCEP in LC-MS-grade water (pH adjusted to 7.0 prior to addition). Acetic acid was added to a final concentration of ~5% and the dilution was centrifuged at 18,000 *g*, r.t for 10 min prior to analytical RP-HPLC and/or LC-MS analyses. Upon completion (based on analytical RP-HPLC and LC-MS), MPAA was removed by dialyzing against ligation buffer using a 3.5 kDa MWCO cassette to enable one-pot Acm removal. For the synthesis of D-CtxB, this reaction was performed as described, but with the corresponding D-peptides.

SUPPORTING INFORMATION

Acm Removal of Peptide 6

The Acm removal was performed as previously reported.[36] Briefly, 200 mM $PdCl_2$ solution was prepared in degassed ligation buffer and added to the peptide solution to a final concentration of 10 mM $PdCl_2$. The reaction was briefly mixed with vortexing prior to adjusting the pH of the reaction to 7.0. The reaction was degassed, and placed on the rotisserie at 37 °C until complete. Time points of the reaction were taken by treating 20 µL with 20 µL of 500 mM DTT (prepared in ddH_20, pH 7.0) for 10 min at r.t. to quench the Pd. Time points were then diluted with 180 µL 20% RP-HPLC buffer B. After vortexing, centrifugation was performed at 18,000 *g*, r.t. for 10 min prior to analytical RP-HPLC and LC-MS analyses. Once Acm removal was complete, the final product peptide 7 was purified by semi-preparative RP-HPLC. For the synthesis of D-CtxB, this reaction was performed as described, but with the corresponding D-peptide.

Folding of Synthetic L- and D-CtxB

Lyophilized full-length peptide 7 was dissolved in denaturing buffer (6 M GnHCl, 50 mM phosphate, pH 7.0) and adjusted to a concentration of 10 µM. The solution was transferred to a 3.5 kDa MWCO dialysis cassette for step-wise dialysis into the following solutions at 4 °C:

- Overnight step into 6 M GnHCl, 50 mM phosphate, 2% DMSO, pH 8.0, 200-fold volume of dialysate to sample
- 4 h step into 3 M GnHCl, 50 mM phosphate, pH 7.4, 200-fold volume of dialysate to sample
- 4 h step into 1 M GnHCl, 50 mM phosphate, pH 7.4, 200-fold volume of dialysate to sample
- Overnight step into 0.5 M GnHCl, 50 mM phosphate, pH 7.4, 200-fold volume of dialysate to sample
- 4 h step into PBS (137 mM NaCl, 2.7 mM KCl, 8 mM Na_2HPO_4, 2 mM KH_2PO_4, pH 7.4), 200-fold volume of dialysate to sample
- 4 h step into PBS, 200-fold volume of dialysate to sample
- Overnight step into PBS, 200-fold volume of dialysate to sample

Folded L-CtxB was clarified by centrifugation at 4000 xg for 15 min at 4 °C prior to size-exclusion chromatography (SEC) to remove soluble aggregates (likely from material with synthetic defects). SEC was completed at 4 °C using a Cytiva Äkta Go instrument with a Cytiva Superdex 75 Increase 10/300 GL column. Using a flow rate of 0.4 mL/min and a mobile phase of PBS, 200 µL of synthetic material was loaded onto the column.

Saved fractions were from the main peak, with an emphasis on avoiding aggregate material. Fractions were pooled, concentrated by centrifugation using a 10 kDa MWCO PES concentrator (Thermo Scientific), and run again on SEC under identical conditions. After two SEC passes, synthetic material was considered pure.Pooled SEC fractions were combined and concentrated by centifugation using a 10 kDa MWCO protein concentrator prior to further biochemical characterizations. Folding of D-CtxB was achieved using the same protocol described above, but with the corresponding full-length D-peptide 7.

Recombinant CtxB Expression and Purification

A pET-IDT vector containing CtxB with a GS linker and a 6xHis-tag on its C-terminus (Integrated DNA Technologies) was used in SHuffle T7 *E. coli* cells (New England Biolabs). Transformation was completed according to cell manufacturer protocols. Briefly, 3 µL of plasmid at ~50 ng/µL was added to 25 µL of SHuffle cells and incubated on ice for 30 min. Next, cells were placed at 42 °C for 30 sec. and then on ice for 5 min. 950 µL of SOC media (Invitrogen) was added before shaking at 250 rpm for 1 h at 37 °C. 250 µL of this was then streaked on kanamycin selection plates. Expression was completed using autoinduction as described by Studier.[39] Autoinduction media was made containing 1% (v/w) tryptone, 0.5% (v/w) yeast extract, 1 mM $MgSO_4$, 1% 1000x metals mix (Teknova), 0.5% glycerol, 0.2% alpha-lactose, 0.05% glucose,100 mM PO_3, 25 mM SO_4, 50 mM NH_4, 100 mM Na, 50 mM K, and kanamycin to 50 mg/L. A colony from the selection plate was added to 500 mL of autoinduction media in a 2.8 L baffled Erlenmeyer flask to shake at 250 rpm for ~18 h at 37 °C. Cells were then pelleted by centrifugation at 12,000 g for 20 min at 4 °C.

The pellet was then resuspended in lysis buffer (50 mM Tris, 250 mM NaCl, 40 mM imidazole, 100 nM phenylmethylsulfonyl fluoride, ~50 mg/L DNAse I, pH 8) and sonicated on ice at 80% power with 6 sec on, 6 sec off cycles for 6 min using a Q500 Qsonica sonicator. Lysate was centrifuged at 4500 g for 20 min at 4 °C and supernatant was then loaded onto a gravity purification column containing Ni-NTA agarose beads (Qiagen) pre-incubated with recombinant wash buffer (50 mM Tris, 250 mM NaCl, 40 mM imidazole, pH 8). Beads were washed twice with 5x bead volume of wash buffer. Protein was eluted with buffer containing 50 mM Tris, 250 mM NaCl, and 400 mM imidazole. Sample was then concentrated using a PES membrane concentrator with a 30,000 Da molecular weight cut-off (Thermo Scientific).

Sample was then further purified using size-exclusion chromatography (SEC) on a Cytiva Äkta Go instrument with a Cytiva Superdex 75 Increase 10/300 GL column. All SEC occurred in a 4 °C room. The mobile phase, PBS, was run at 0.4 mL/min. Fractions under the main peak were collected and then re-concentrated using the above concentrators. Protein concentration was established as described for peptides.

Circular Dichroism Analysis of L- and D-CtxB

CD spectra for folded synthetic L-CtxB and D-CtxB, as well as recombinant CtxB (reference spectrum), were obtained using a JASCO J-810 circular dichroism spectrometer or an AVIV model 410 circular dichroism spectrometer. Samples (20 μM protein in PBS - 137 mM NaCl, 2.7 mM KCl, 8 mM Na_2HPO_4, 2 mM KH_2PO_4, pH 7.4) and blanks (PBS) were added to a 1 mm quartz cuvette and analyzed at 25°C. Wavelength scans from 204-260 nm were performed in triplicate at 2 nm resolution with 1 second averaging time. Scans were averaged and blank-subtracted before normalization to mean residue molar ellipticity ($[\theta] = 10^6 * \theta/(C * l * n)$, where C is concentration of protein in μM, l is path length in millimeters, and n is the number of peptide bonds in the protein). The dynode voltage from 204-260 nm was monitored throughout CD experiments and did not exceed 500 volts.

Analytical Size-Exclusion Chromatography Analysis of L- and D-CtxB

Analytical size-exclusion chromatography (SEC) was completed using a Phenomenex Yarra 3 µm SEC-2000 150 mm x 4.6 mm column on an Agilent 1260 Infinity II with a diode array detector. To mitigate an observed His6 interaction with the column with the recombinant CtxB sample, all samples were run in high salt (300 mM NaCl) PBS at 0.45 mL/min. All runs used 6 µL injections of protein at 20 µM. SEC standards were Bio-Rad Gel Filtration Standards and were diluted according to manufacturer recommendations.

Binding ELISA of L-CtxB with GM1

Flat-well MaxiSorp 96-well plates (Thermo Scientific) were coated with either 100 µL of 0.0125 ng/µL GM1 (Monosialoganglioside GM1 from bovine brain, Sigma Aldrich) in PBS or just PBS and set to shake overnight at 4 °C. Plates were then washed three times with 300 µL wash buffer (25 mM Tris, 150 mM NaCl, 0.05% Tween-20, pH 8). Next, 200 µL blocking buffer (5% w/v Biorad Blocking Buffer in wash buffer) was added to each well with shaking at r.t. for 1 h. A wash was completed as described above. Relevant concentrations of biotinylated synthetic CtxB and recombinant CtxB were mixed to a total volume of 100 µL and then added to respective

wells to shake for 1 h at r.t. Plates were washed as described above. 100 µL of 1:5000 dilution of horseradish peroxidase-conjugated streptavidin (Thermo Fisher Scientific) was added to each well to shake for 1 h at r.t. Plates were washed again as described above. 100 µL of 3,3′,5,5′-Tetramethylbenzidine (TMB) at r.t. was added to each well and incubated for 14 min before quenching with 100 µL of 1 N HCl. Plate absorbance was read at 450 nm.

SECTION 3: Supplementary Figures

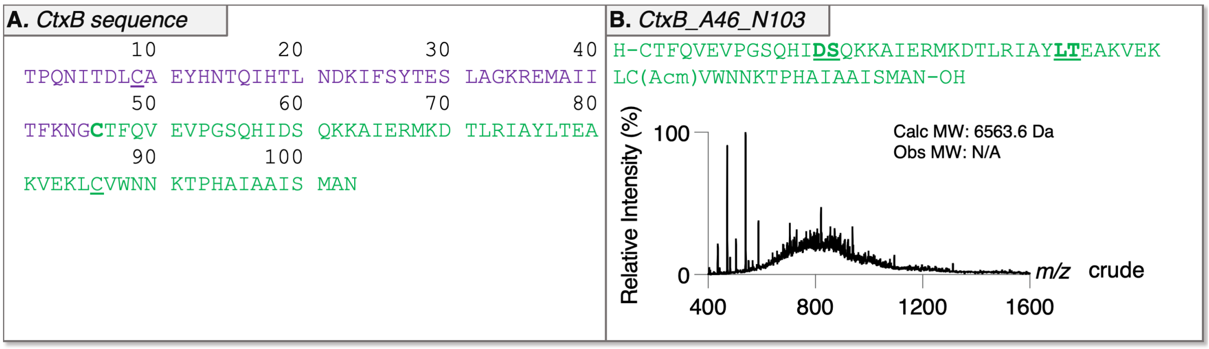

Figure S1. SPPS attempt for the synthesis of CtxB using a two-segment synthesis approach. (A) Sequence of CtxB depicting the two-segment synthesis approach. Bolded residue indicates the incorporation of a non-native Cys for NCL. Underlined residues indicate Cys protected with Acm. (B) Failed SPPS attempt of CtxB C-terminal half for the two-segment approach with the G45-A46 NCL junction. The peptide was synthesized at a 25 µmol scale and cleaved under 25 µmol scale conditions with EDT. The synthesis was deemed unsuccessful by LC-MS analysis using method A. MS from the entire LC-MS chromatogram is reported. Bolded and underlined residues indicate the use of pseudoprolines during SPPS. Met residues were subsituted with Nle.

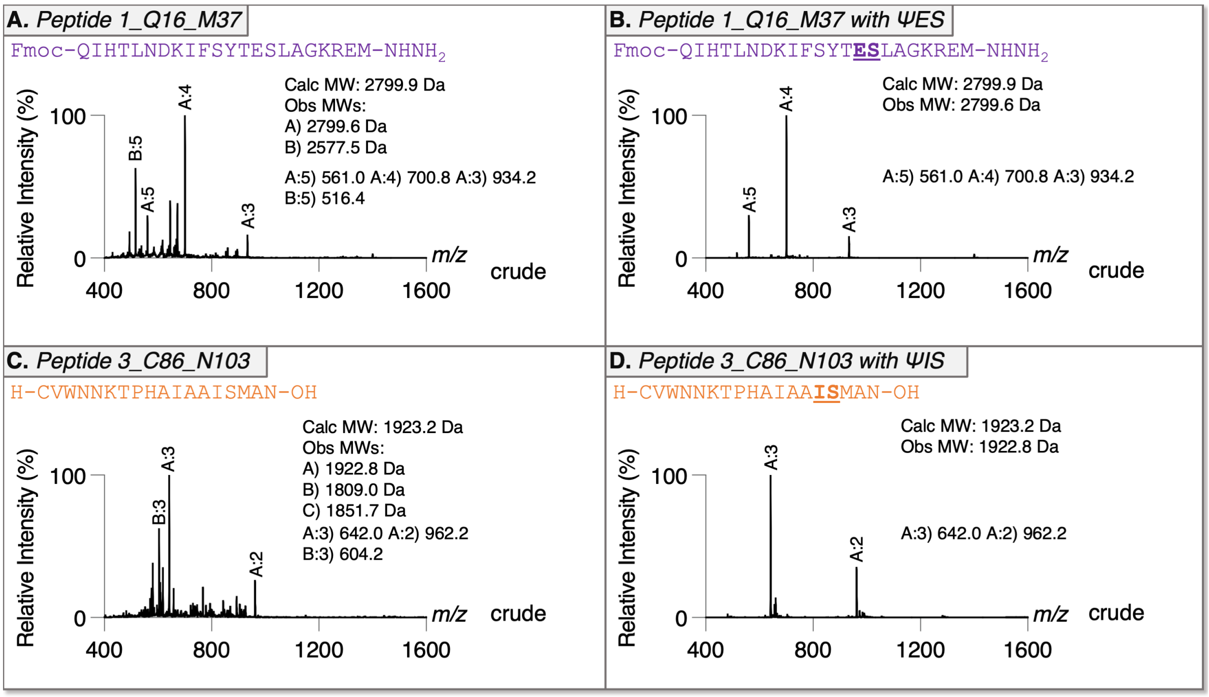

Figure S2. Solid-phase peptide synthesis optimization of peptide **1** and peptide **3** with pseudoproline dipeptides. A) Crude peptide MS analysis of the C-terminal half (Q16-M37) of peptide **1** without pseudoproline dipeptides. B) Crude peptide MS analysis of the C-terminal half (Q16-M37) of peptide **1** with ΨES (29-30) which improved crude peptide quality. C) Crude peptide MS analysis of peptide **3** without pseudoproline dipeptides. D) Crude peptide MS analysis of peptide **3** with ΨIS (99-100) which improved crude peptide quality. Peptide **1** was synthesized at a 25 μmol scale and cleaved under 25 μmol scale conditions. Peptide **3** was synthesized at a 25 μmol scale and cleaved under 25 μmol scale conditions with EDT. LC-MS analysis was conducted using method A. MS from the entire LC-MS chromatogram is reported. Bolded and underlined residues indicate the use of pseudoprolines during SPPS. Italicized residues were double coupled during SPPS. Met residues were subsituted with Nle.

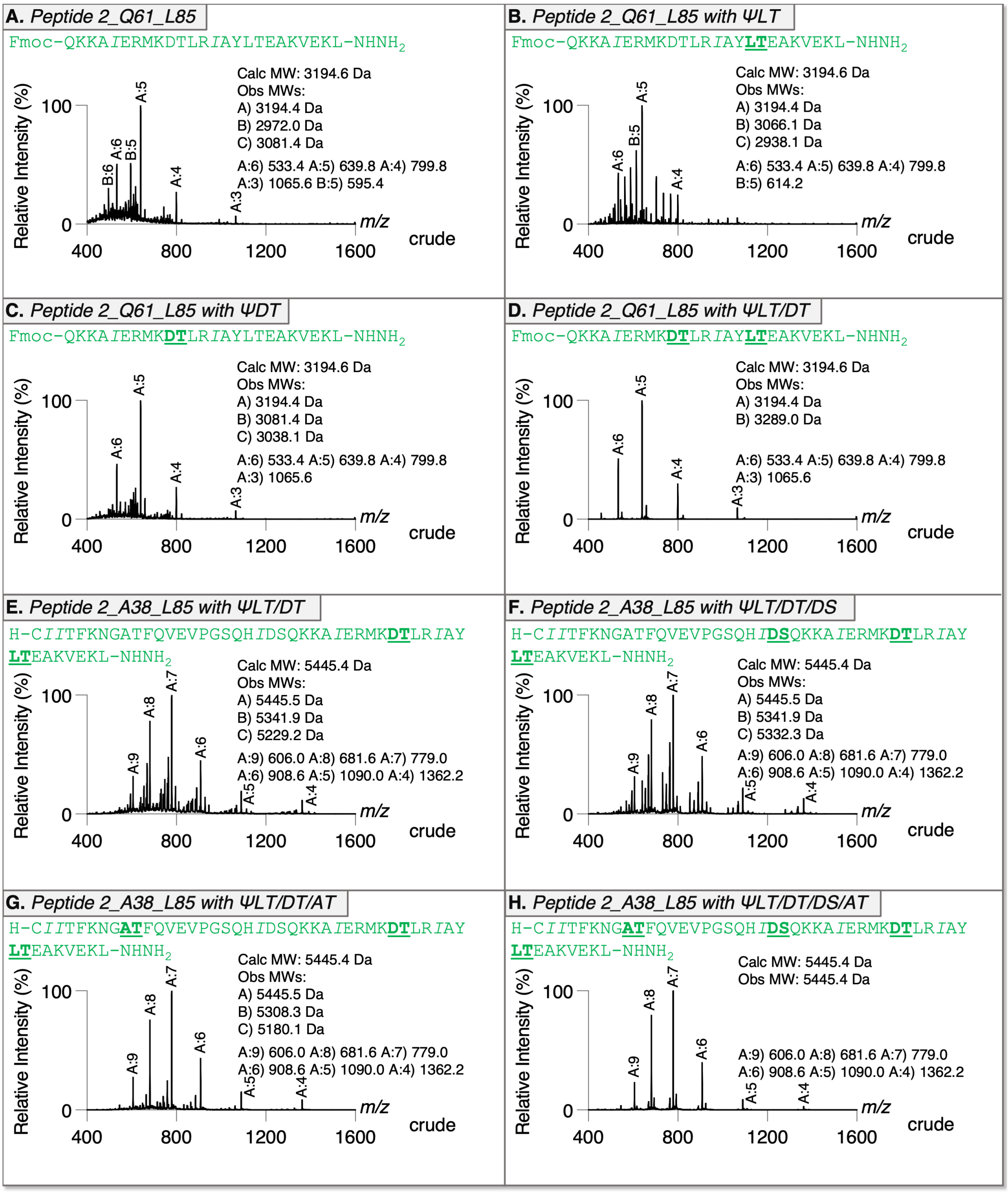

Figure S3. Solid-phase peptide synthesis optimization of peptide **2**. A) Crude peptide MS analysis of the C-terminal half (Q61-L85) of peptide **2** without pseudoproline dipeptides. B) Crude peptide MS analysis of the C-terminal half (Q61-L85) of peptide **2** with ΨLT (77-78). C) Crude peptide MS analysis of the C-terminal half (Q61-L85) of peptide **2** with ΨDT (70-71). D) Crude peptide MS analysis of the C-terminal half (Q61-L85) of peptide **2** with ΨLT (77-78) and ΨDT (70-71), which improved crude peptide quality. E) Crude peptide MS analysis of peptide **2** with ΨLT (77-78) and ΨDT (70-71). F) Crude peptide MS analysis of peptide **2** with ΨLT (77-78), ΨDT (70-71), and ΨDS (59-60). G) Crude peptide MS analysis of peptide **2** with ΨLT (77-78), ΨDT (70-71), and ΨAT (46-47). H) Crude peptide MS analysis of peptide **2** with ΨLT (77-78), ΨDT (70-71), ΨDS (59-60), and ΨAT (46-47), which improved crude peptide quality. Peptide **2** was synthesized at a 25 µmol scale and cleaved under 25 µmol scale conditions with EDT. LC-MS analysis was conducted using method A. MS from the entire LC-MS chromatogram is reported. Bolded and underlined residues indicate the use of pseudoprolines during SPPS. Italicized residues were double coupled during SPPS. Met residues were subsituted with Nle.

SUPPORTING INFORMATION

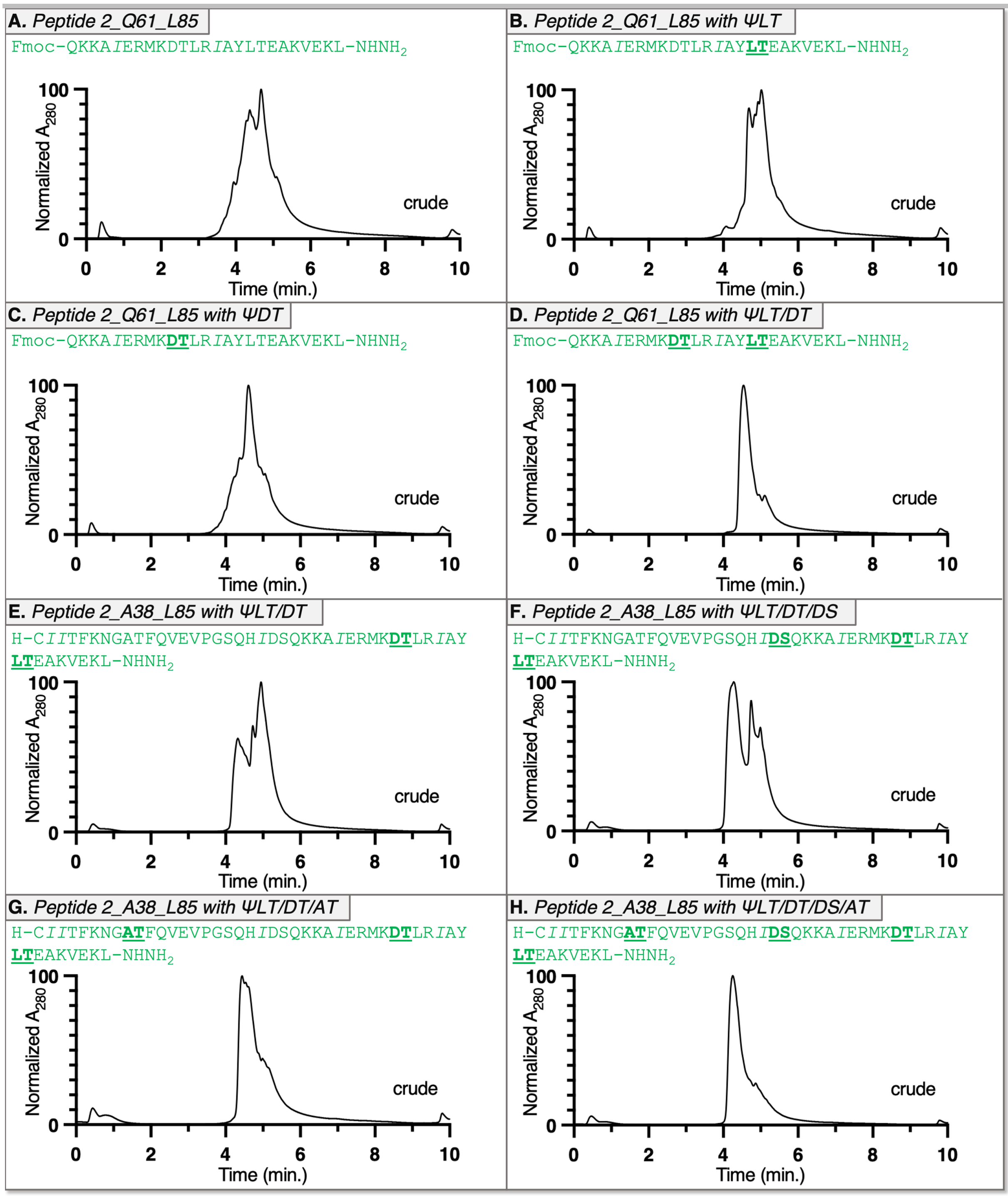

Figure S4. Solid-phase peptide synthesis optimization of peptide **2**. Normalized A_{280} signals from each respective LC-MS analysis in Figure S3 (A-H).

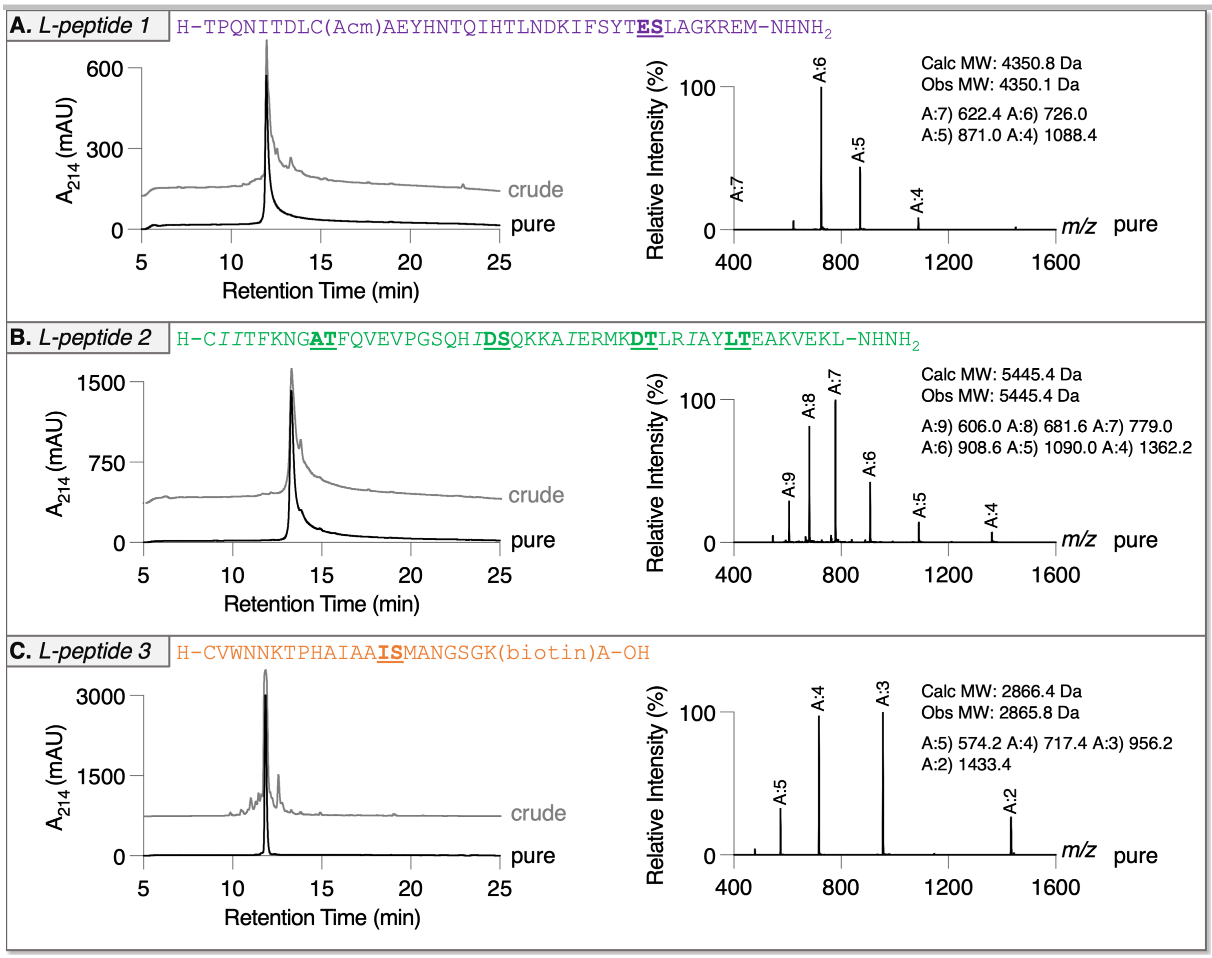

Figure S5. L-peptides for the chemical synthesis of biotinylated L-CtxB. (A) L-peptide **1** was synthesized at 25 µmol scale, cleaved under standard 25 µmol scale conditions, and purified using purification method A with an isolated yield of 19%. (B) L-peptide **2** was synthesized at 25 µmol scale, cleaved under 25 µmol scale conditions with EDT, and purified using purification method B with an isolated yield of 20%. (C) L-peptide **3** was synthesized at 25 µmol scale, cleaved under 25 µmol scale conditions with EDT, and purified using purification method C with an isolated yield of 57%. Analytical RP-HPLC method A and LC-MS method A were used for the analyses of pure and crude peptides. MS of pure peptides taken from the entire LC-MS chromatograms are reported. Bolded and underlined residues indicate the use of pseudoprolines during SPPS. Italicized residues were double coupled during SPPS. Met residues were subsituted with Nle.

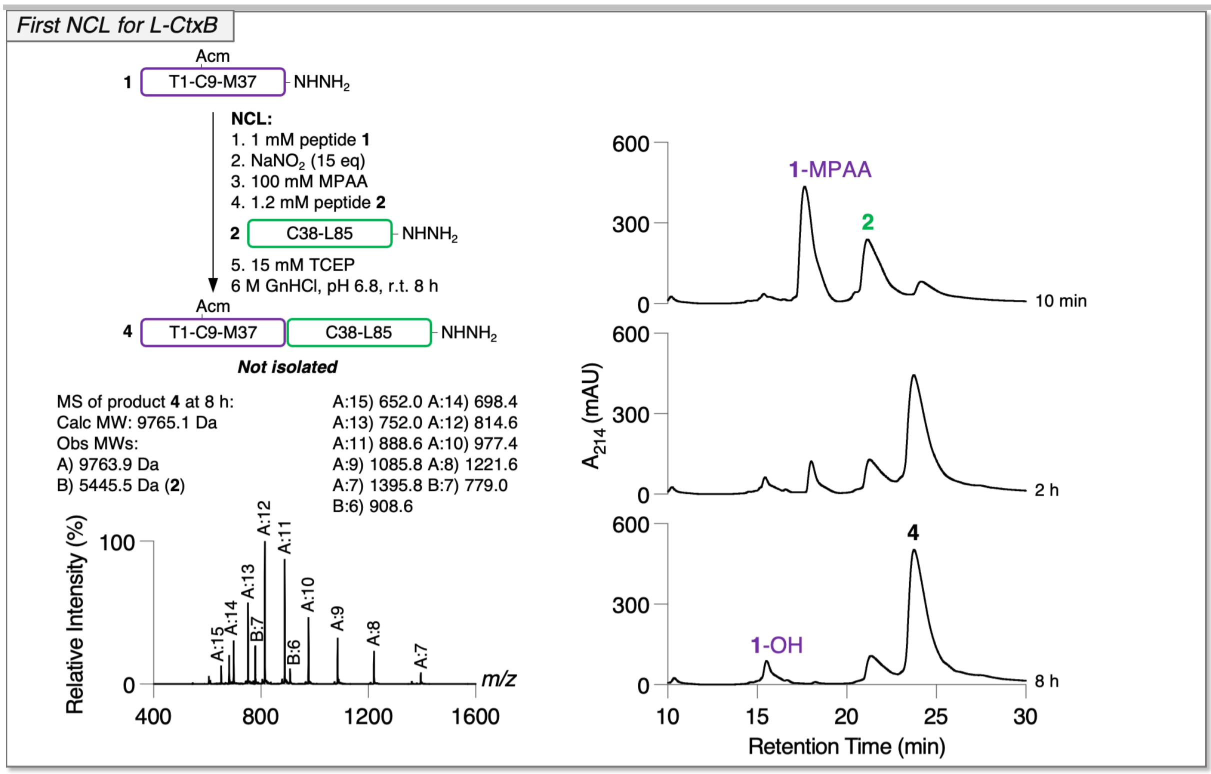

Figure S6. First NCL for the chemical synthesis of L-CtxB. L-peptide **1** (Fig S4A) was ligated to L-peptide **2** (Fig S4B) via peptide-$NHNH_2$ activation by $NaNO_2$ treatment at pH 3.0 and MPAA thiolysis. The reaction was initiated by adjusting the pH to 6.8 after which TCEP was added. Depletion of L-peptide **1**-MPAA thioester was observed at 8 h resulting in conversion to ligated L-segment **4**. Analytical RP-HPLC method B and LC-MS method B were used for the analyses. MS of the individually labeled peak of L-segment **4** from the analytical RP-HPLC chromatogram is reported. Thioester hydrolysis is indicated with -OH. Met residues were subsituted with Nle. The analytical RP-HPLC traces of the NCL time points are also shown in Fig 2A.

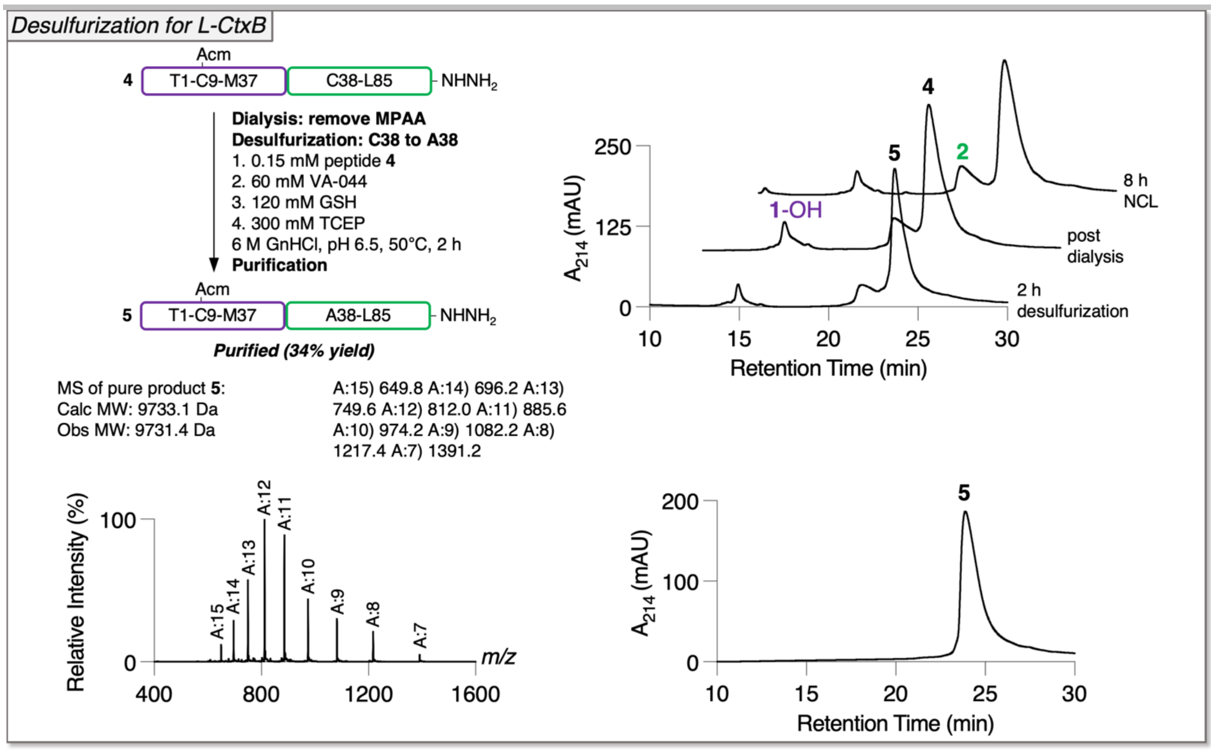

Figure S7. Desulfurization reaction for the chemical synthesis of L-CtxB. Ligated L-segment **4** (Fig S5) was dialyzed into desulfurization buffer to remove MPAA and desulfurization was achieved with 60 mM VA-044, 120 mM GSH, and 300 mM TCEP at 50 °C. The reaction reached completion at 2 h after which L-product **5** was purified by semi-preparative RP-HPLC using purification method D with a 34% isolated yield over two reactions done in one pot. Analytical RP-HPLC method B and LC-MS method B were used for the analyses. MS of pure L-product **5** from the entire LC-MS chromatogram is reported. Met residues were subsituted with Nle. The analytical RP-HPLC chromatogram of purified desulfurized ligated L-segment **5** is also shown in Fig 2B.

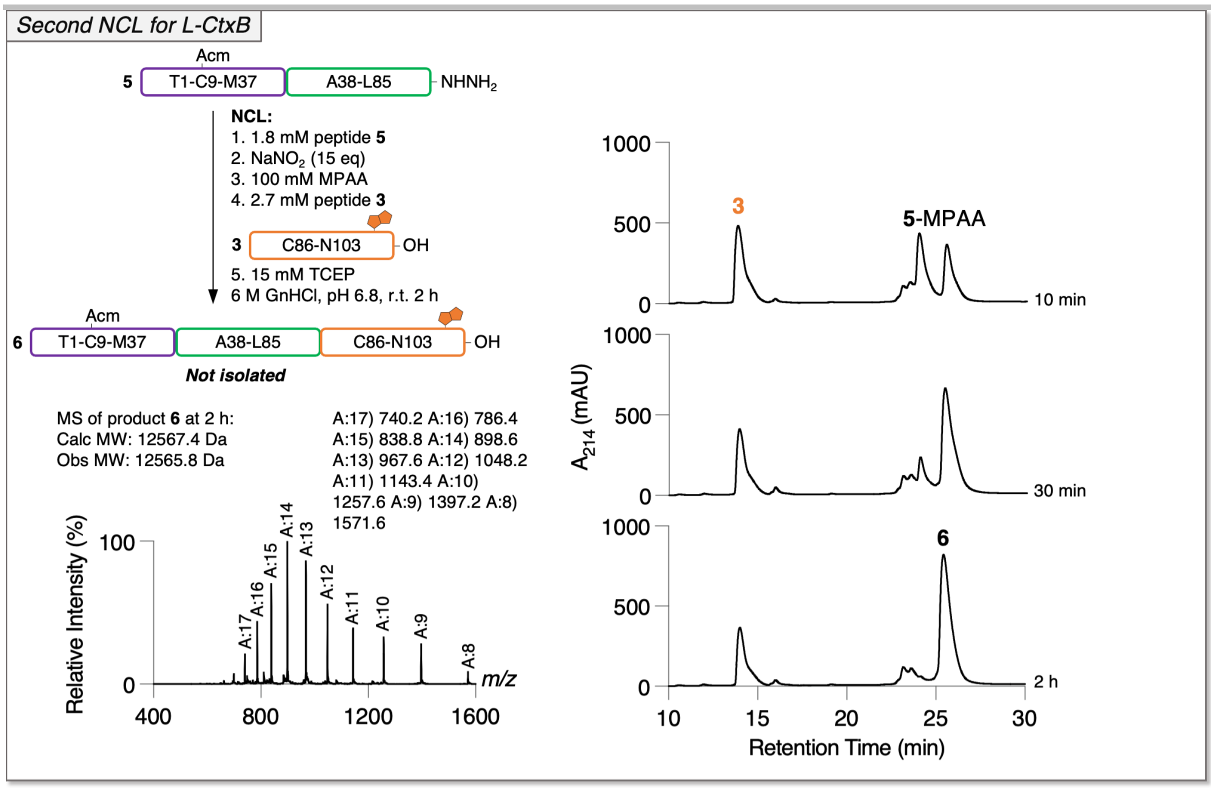

Figure S8. Second NCL for the chemical synthesis of L-CtxB. L-peptide **5** (Fig S6) was ligated to L-peptide **3** (Fig S4C) via peptide-$NHNH_2$ activation by $NaNO_2$ treatment at pH 3.0 and MPAA thiolysis. The reaction was initiated by adjusting the pH to 6.8 after which TCEP was added. Depletion of L-peptide **5**-MPAA thioester was observed at 2 h resulting in conversion to ligated L-product **6**. Analytical RP-HPLC method C and LC-MS method B were used for the analyses. MS of the individually labeled peak of L-product **6** from the analytical RP-HPLC chromatogram is reported. Met residues were subsituted with Nle. The analytical RP-HPLC traces of the NCL time points are also shown in Fig 2A.

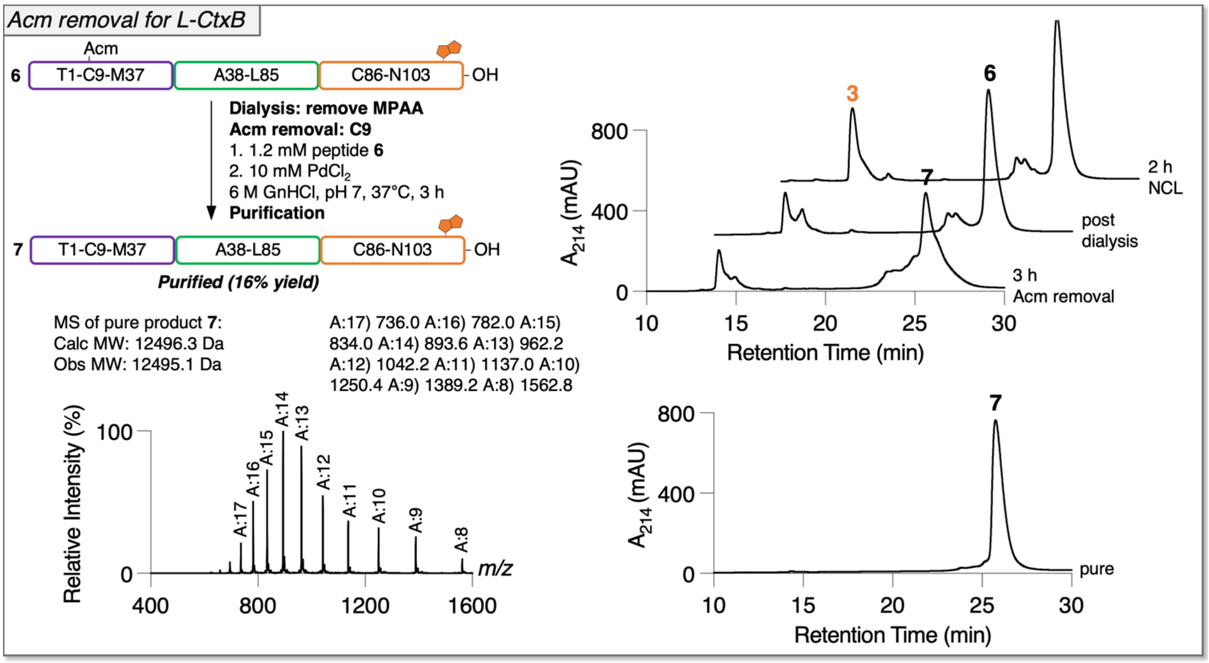

Figure S9. Acm removal reaction for the chemical synthesis of L-CtxB. Ligated L-product **6** (Fig S7) was dialyzed into ligation buffer to removed MPAA and the reaction was treated with 10 mM $PdCl_2$ for 3 h at 37 °C. The Pd was

quenched with 250 mM DTT and the reaction was purified by semi-preparative RP-HPLC using purification method E to isolate L-CtxB **7** with 16% isolated yield over two reactions done in one pot. Analytical RP-HPLC method C and LC-MS method B were used for the analyses. MS of pure L-CtxB **7** from the entire LC-MS chromatogram is reported. Met residues were subsituted with Nle. The analytical RP-HPLC chromatogram of L-CtxB **7** is also shown in Fig 2C.

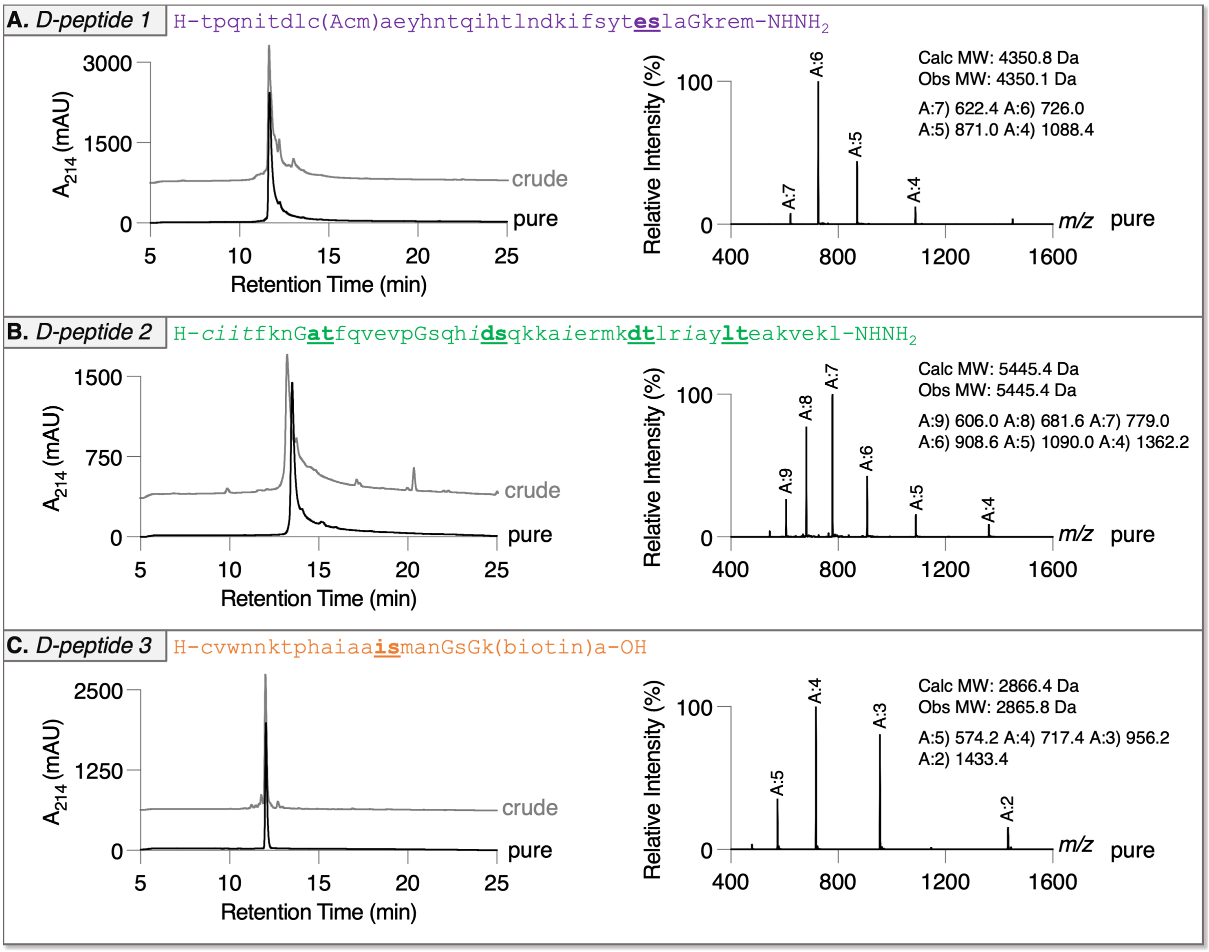

Figure S10. D-peptides for the chemical synthesis of biotinylated D-CtxB. (A) D-peptide **1** was synthesized at 25 µmol scale, cleaved under standard 25 µmol scale conditions, and purified using purification method A with an isolated yield of 20%. (B) D-peptide **2** was synthesized at 25 µmol scale, cleaved under 25 µmol scale conditions with EDT, and purified using purification method B with an isolated yield of 18%. (C) D-peptide **3** was synthesized at 25 µmol scale, cleaved under 25 µmol scale conditions with EDT, and purified using purification method C with an isolated yield of 47%. Analytical RP-HPLC method A and LC-MS method A were used for the analyses of pure and crude peptides. MS of pure peptides taken from the entire LC-MS chromatograms are reported. Bolded and underlined residues indicate the use of pseudoprolines during SPPS. Italicized residues were double-coupled during SPPS. Met residues were subsituted with Nle.

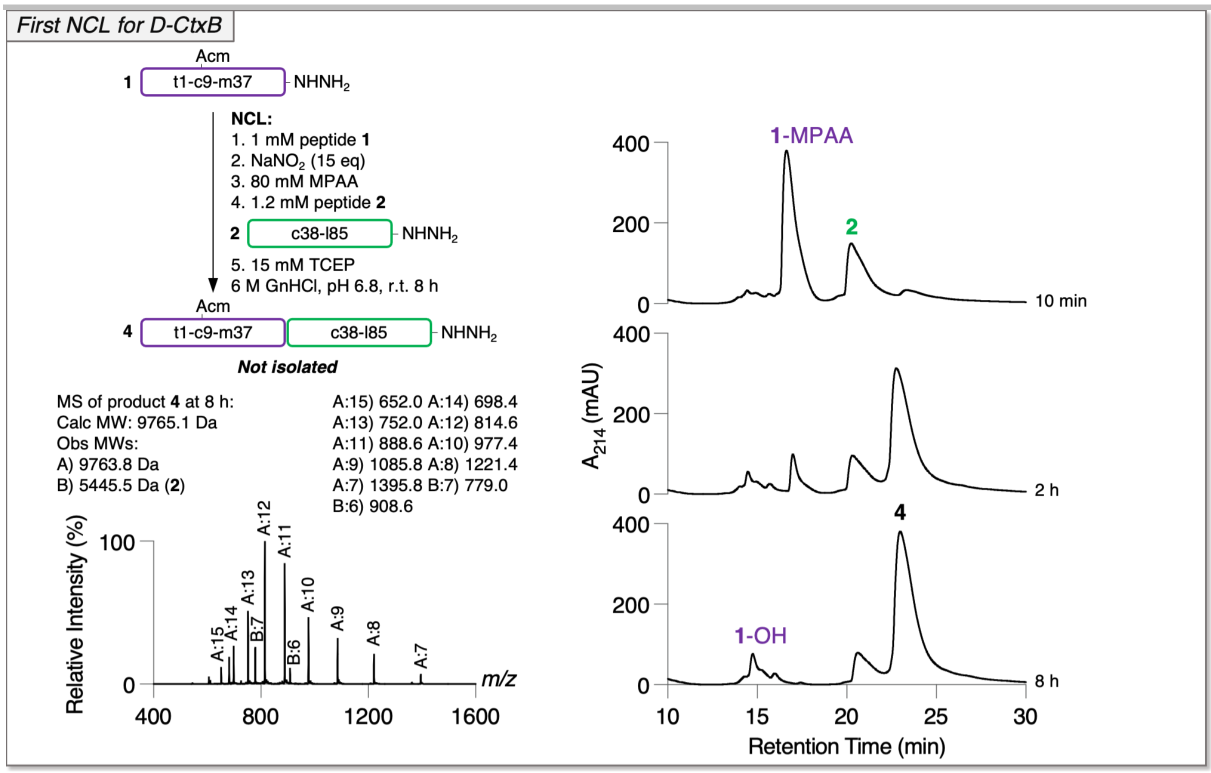

Figure S11. First NCL for the chemical synthesis of D-CtxB. D-peptide **1** (Fig S9A) was ligated to D-peptide **2** (Fig S9B) via peptide-$NHNH_2$ activation by $NaNO_2$ treatment at pH 3.0 and MPAA thiolysis. The reaction was initiated by adjusting the pH to 6.8 after which TCEP was added. Depletion of D-peptide **1**-MPAA thioester was observed at 8 h resulting in conversion to ligated D-segment **4**. Analytical RP-HPLC method B and LC-MS method B were used for the analyses. MS of the individually labeled peak of D-segment **4** from the analytical RP-HPLC chromatogram is reported. Thioester hydrolysis is indicated with -OH. Met residues were subsituted with Nle.

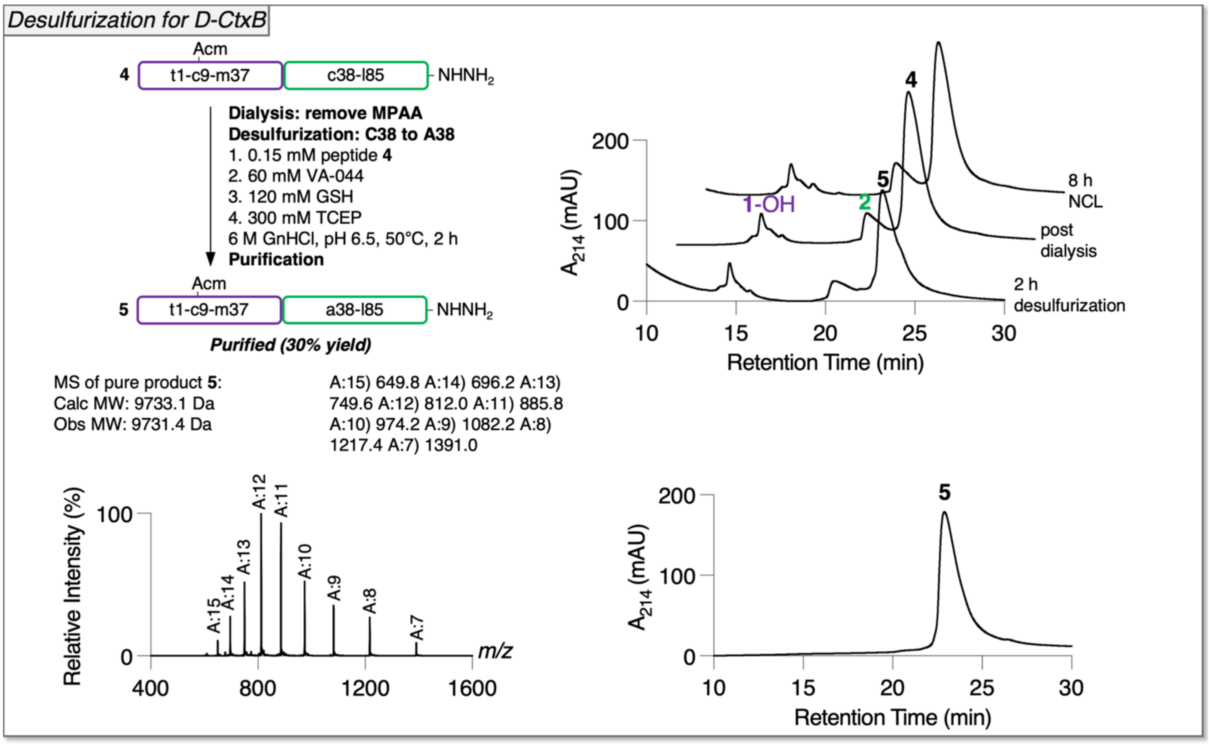

Figure S12. Desulfurization reaction for the chemical synthesis of D-CtxB. Ligated D-segment **4** (Fig S10) was dialyzed into desulfurization buffer to removed MPAA and desulfurization was achieved with 60 mM VA-044, 120 mM GSH, and 300 mM TCEP at 50 °C. The reaction reached completion at 2 h after which D-product **5** was purified by semi-preparative RP-HPLC using purification method D with a 30% isolated yield over two reactions done in one pot. Analytical RP-HPLC method B and LC-MS method B were used for the analyses. MS of pure D-product **5** from the entire LC-MS chromatogram is reported. Met residues were subsituted with Nle. The analytical RP-HPLC chromatogram and MS of purified desulfurized ligated D-segment **5** are also shown in Fig 2B.

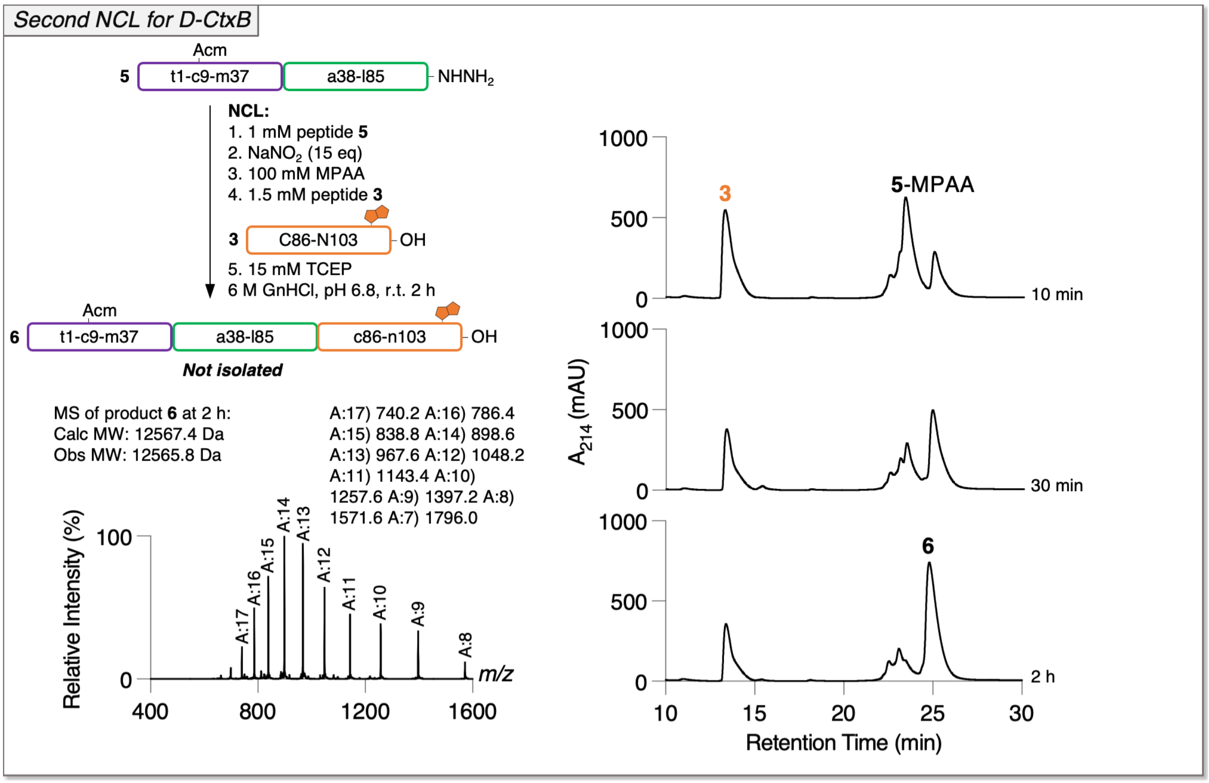

Figure S13. Second NCL for the chemical synthesis of D-CtxB. D-peptide **5** (Fig S11) was ligated to D-peptide **3** (Fig S9C) via peptide-$NHNH_2$ activation by $NaNO_2$ treatment at pH 3.0 and MPAA thiolysis. The reaction was initiated by adjusting the pH to 6.8 after which TCEP was added. Depletion of D-peptide **5**-MPAA thioester was observed at 2 h resulting in conversion to ligated D-product **6**. Analytical RP-HPLC method C and LC-MS method B were used for the analyses. MS of the individually labeled peak of D-product **6** from the analytical RP-HPLC chromatogram is reported. Met residues were subsituted with Nle.

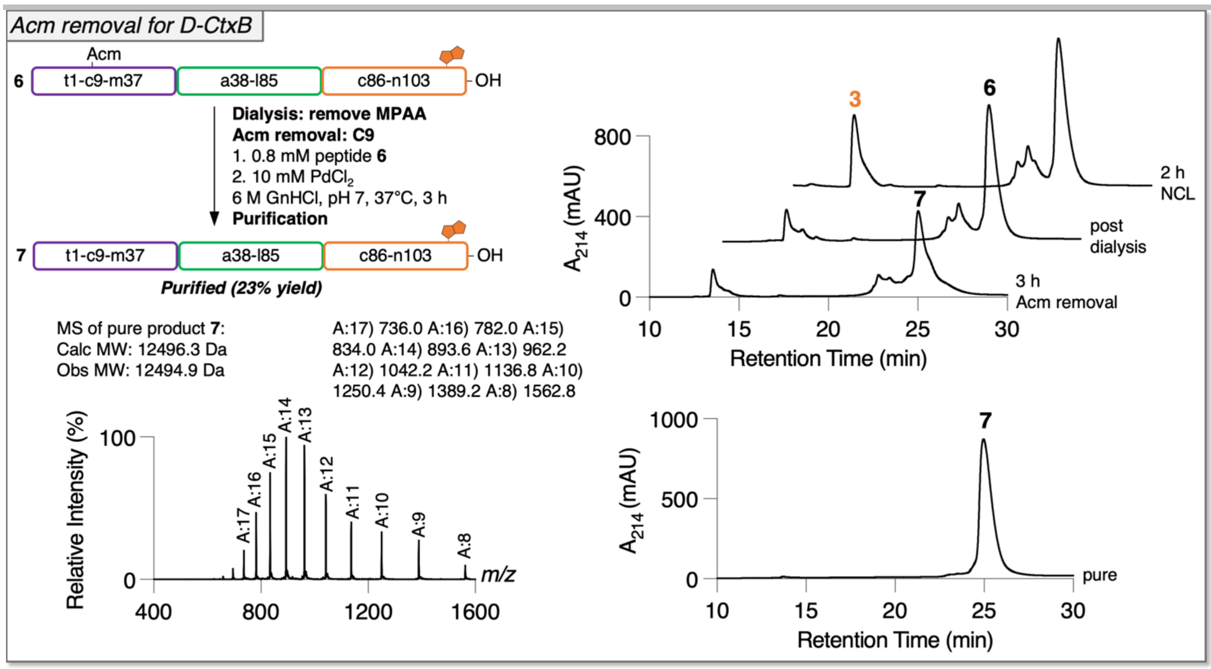

Figure S14. Acm removal reaction for the chemical synthesis of D-CtxB. Ligated D-product **6** (Fig S12) was dialyzed into ligation buffer to removed MPAA and the reaction was treated with 10 mM $PdCl_2$ for 3 h at 37 °C. The Pd was quenched with 250 mM DTT and the reaction was purified by semi-preparative RP-HPLC using purification method E to isolate D-CtxB **7** with 23% isolated yield over two reactions done in one pot. Analytical RP-HPLC method C and LC-MS method B were used for the analyses. MS of pure D-CtxB **7** from the entire LC-MS chromatogram is reported. Met residues were subsituted with Nle. The analytical RP-HPLC chromatogram and MS of D-CtxB **7** are also shown in Fig 2C.

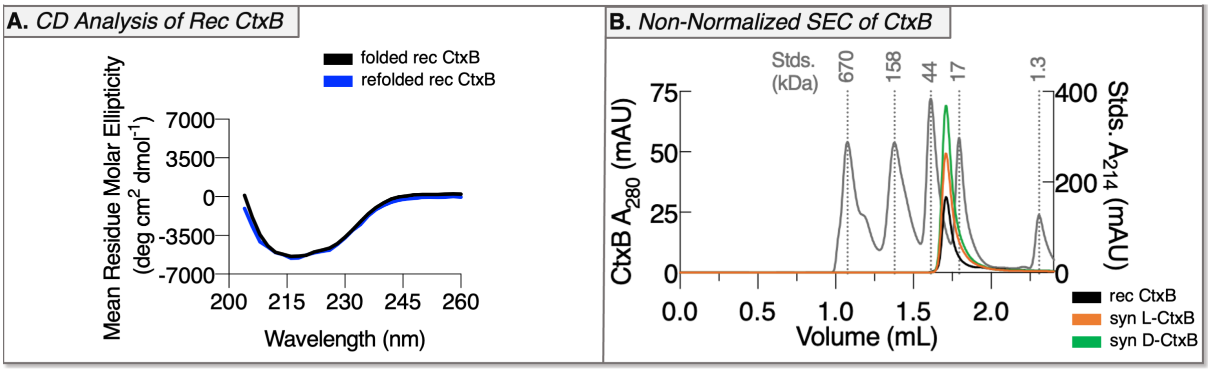

Figure S15 CD of recombinant CtxB and SEC of L-, D-, and recombinant SEC. (A) Overlayed CD spectrum of folded recombinant CtxB and re-folded product after denaturing show a successful folding process. (B) Non-normalized SEC of CtxB constructs shows diminished peak integration for recombinant samples compared to synthetic samples. Each sample had a 6 µL injection at 20 µM. Bio-Rad gel filtration standards were used according to manufacturer recommendations.

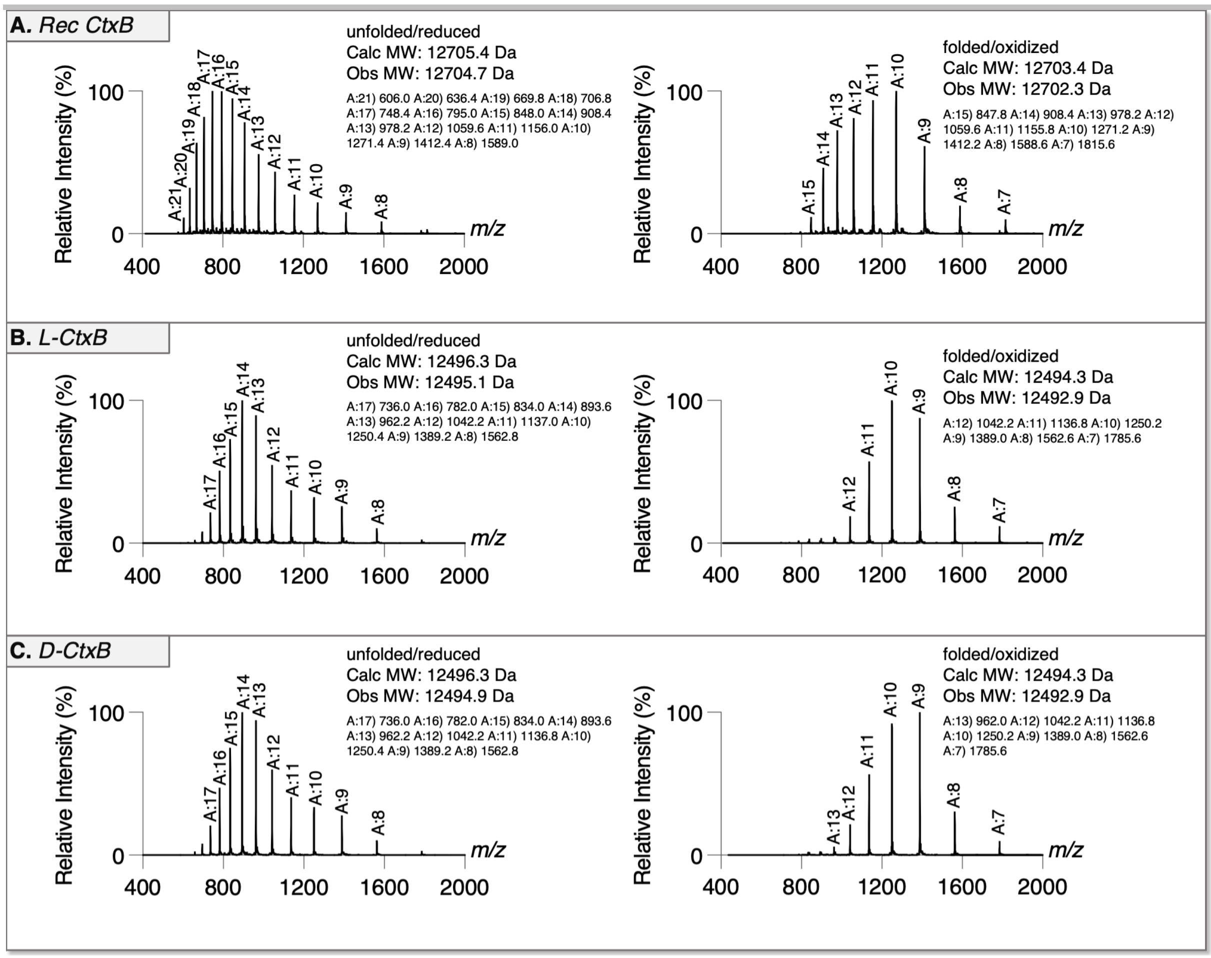

Figure S16. MS analysis of unfolded and folded CtxB constructs. (A) Recombinant (Rec) CtxB before and after refolding with the expected -2 Da mass change from successful disulfide formation. (B) Synthetic L-CtxB (Fig S8) before and after folding with the expected -2 Da mass change from successful disulfide formation. (C) Synthetic D-CtxB (Fig S13) before and after folding with the expected -2 Da mass change from successful disulfide formation. LC-MS method B was used for the analysis.
